# The Combined Deficiency of Immunoproteasome Subunits Affects Both the Magnitude and Quality of Pathogen- and Genetic Vaccination-Induced CD8^+^ T Cell Responses to the Human Protozoan Parasite *Trypanosoma cruzi*

**DOI:** 10.1371/journal.ppat.1005593

**Published:** 2016-04-29

**Authors:** Jonatan Ersching, José R. Vasconcelos, Camila P. Ferreira, Braulia C. Caetano, Alexandre V. Machado, Oscar Bruna–Romero, Monique A. Baron, Ludmila R. P. Ferreira, Edécio Cunha-Neto, Kenneth L. Rock, Ricardo T. Gazzinelli, Maurício M. Rodrigues

**Affiliations:** 1 Centro de Terapia Celular e Molecular and Departamento de Microbiologia, Imunologia e Parasitologia, Universidade Federal de São Paulo - Escola Paulista de Medicina, São Paulo, São Paulo, Brazil; 2 Departamento de Biociências, Universidade Federal de São Paulo, Santos, São Paulo, Brazil; 3 Departments of Medicine and Pathology, University of Massachusetts Medical School, Worcester, Massachusetts, United States of America; 4 Centro de Pesquisas René Rachou, FIOCRUZ, Belo Horizonte, Minas Gerais, Brazil; 5 Departamento de Microbiologia, Imunologia e Parasitologia, Universidade Federal de Santa Catarina, Florianópolis, Santa Catarina, Brazil; 6 Instituto do Coração (InCor), Faculdade de Medicina - Universidade de São Paulo, São Paulo, São Paulo, Brazil; 7 Universidade Santo Amaro, São Paulo, São Paulo, Brazil; 8 Departamento de Bioquímica e Imunologia, Instituto de Ciências Biológicas, Universidade Federal de Minas Gerais, Belo Horizonte, Minas Gerais, Brazil; Imperial College London, UNITED KINGDOM

## Abstract

The *β1i*, *β2i* and *β5i* immunoproteasome subunits have an important role in defining the repertoire of MHC class I-restricted epitopes. However, the impact of combined deficiency of the three immunoproteasome subunits in the development of protective immunity to intracellular pathogens has not been investigated. Here, we demonstrate that immunoproteasomes play a key role in host resistance and genetic vaccination-induced protection against the human pathogen *Trypanosoma cruzi* (the causative agent of Chagas disease), immunity to which is dependent on CD8^+^ T cells and IFN-γ (the classical immunoproteasome inducer). We observed that infection with *T*. *cruzi* triggers the transcription of immunoproteasome genes, both in mice and humans. Importantly, genetically vaccinated or *T*. *cruzi*-infected *β1i*, *β2i* and *β5i* triple knockout (TKO) mice presented significantly lower frequencies and numbers of splenic CD8^+^ effector T cells (CD8^+^CD44^high^CD62L^low^) specific for the previously characterized immunodominant (VNHRFTLV) H-2K^b^-restricted *T*. *cruzi* epitope. Not only the quantity, but also the quality of parasite-specific CD8^+^ T cell responses was altered in TKO mice. Hence, the frequency of double-positive (IFN-γ^+^/TNF^+^) or single-positive (IFN-γ^+^) cells specific for the H-2K^b^-restricted immunodominant as well as subdominant *T*. *cruzi* epitopes were higher in WT mice, whereas TNF single-positive cells prevailed among CD8^+^ T cells from TKO mice. Contrasting with their WT counterparts, TKO animals were also lethally susceptible to *T*. *cruzi* challenge, even after an otherwise protective vaccination with DNA and adenoviral vectors. We conclude that the immunoproteasome subunits are key determinants in host resistance to *T*. *cruzi* infection by influencing both the magnitude and quality of CD8^+^ T cell responses.

## Author Summary

CD8^+^ t lymphocytes are cells of the immune system that mediate control of intracellular infections by viruses, prokaryote as well as eukaryote pathogens. To confer protection, these lymphocytes need to be elicited by pathogen peptides that are presented in association with MHC class I molecules. The degradation of self and microbial proteins by catalytic domains of the cytosolic proteasome β1, β2 and β5 subunits is intimately linked to the generation of MHC class I-restricted epitopes, which in turn are important determinants of the kinetics, specificity and efficiency of CD8^+^ T cell-mediated immunity. Importantly, inflammatory stimuli trigger the expression of the inducible alternative β1i, β2i and β5i subunits that form the immunoproteasomes. The qualitative and quantitative importance of immunoproteasomes in generating CD8^+^ T cell epitopes has recently been demonstrated in mice that are simultaneously devoid of the β1i, β2i and β5i subunits. In this study, we explored the role of immunoproteasomes in host resistance to *Trypanosoma cruzi*, a protozoan parasite that causes Chagas disease. We found that β1i, β2i and β5i triply deficient mice have an impaired response of CD8^+^ T cells and are highly susceptible to primary infection with *T*. *cruzi*. We also demonstrated that host resistance induced by a genetic vaccine able to protect normal mice from *T*. *cruzi* challenge fails to do so in the immunoproteasome-deficient mice. Our study provides strong evidences that β1i, β2i and β5i immunoproteasome subunits are important determinants of both the magnitude and quality of CD8^+^ T cell responses as well as immune-mediated host resistance to a human pathogen.

## Introduction

CD8^+^ T cells are important mediators of pathogen control during intracellular infections. Sufficient induction of these cells leads to pathogen elimination [1–8], whereas weak or exacerbated CD8^+^ T cell stimulation may lead to pathology [9–17]. Therefore, the proper induction of CD8^+^ T cells must be tightly regulated and may be co-opted in the development of new vaccines against intracellular pathogens [18–22].

Critical in the process of CD8^+^ T cell induction is the kinetics and efficiency of the provision of MHC class I-restricted epitopes recognized by these lymphocytes, which is linked to the degradation of mature proteins and defective ribosomal products in the cytosol by barrel-shaped structures denoted proteasomes, as recently reviewed [23, 24]. The catalytic activity of proteasomes is attributed to 3 subunits (β1 (Psmb6), β2 (Psmb7), and β5 (Psmb5)) located in each of the two inner β rings of the 20S core. In addition, proteasomes featuring the alternative catalytic subunits β1i (LMP2 or Psmb9), β2i (MECL1 or Psmb10) and β5i (LMP7 or Psmb8) are named immunoproteasomes, which are constitutively expressed in some hematopoietic cells and may be induced by inflammatory stimuli such as IFN-γ, IFN-β or TNF in other cell types (reviewed in [25]).

The immunoproteasomes enhance the quantity and diversity of MHC class I-restricted peptides generated and their consequent impact on the magnitude and breath of protective responses of CD8^+^ T cells against intracellular pathogens has long been studied by several groups. However, the β1i, β2i and β5i proteins have a redundant role, and only partial phenotype is observed in mice lacking a single functional gene encoding one of the immunoproteasome subunits. Only recently a mouse concomitantly defective of all three immunoproteasome genes (TKO mouse) was made available, allowing the observation that immunoproteasomes are more relevant for the repertoire of MHC class I-presented peptides than thought before, so much so that WT splenocytes are rejected by TKO mice [26]. However, the participation of immunoproteasomes in the control of infections by most pathogens studied so far was only incremental.

Here, we evaluated the role of immunoproteasomes in the CD8^+^ T cell-mediated immunity, resistance to infection, and protective immunity conferred by genetic vaccination against *Trypanosoma cruzi*, a human protozoan parasite and causative agent of Chagas disease. Because control of infection by *T*. *cruzi* is critically dependent on CD8^+^ T cells and IFN-γ (reviewed in [27]), we reasoned that the immunoproteasome could be specially relevant for protection in this model. Confirming our hypothesis, we found that CD8^+^ T cell immune responses to *T*. *cruzi* epitopes were remarkably weaker in TKO mice infected with *T*. *cruzi* or immunized with an adenoviral vaccine vector expressing an immunodominant parasite antigen. Not only the quantity, but also the quality of parasite-specific CD8^+^ T cell responses was altered in TKO mice, as indicated by the higher frequency of double-positive (IFN-γ^+^/TNF^+^) or single-positive (IFN-γ^+^) cells specific for immunodominant as well as subdominant *T*. *cruzi* epitopes in WT versus TKO mice. Finally, another highly relevant finding was that both *naïve* and vaccinated TKO mice were extremely susceptible to experimental infection, with most animals succumbing to an otherwise non-lethal challenge. These observations establish that immunoproteasomes play a critical role in the generation of immunogenic peptides and the development of protective *T*. *cruzi-*specific CD8^+^ T lymphocytes.

## Results

### Reduced antigen presentation of MHC class I-restricted *T*. *cruzi* epitopes by immunoproteasome-deficient dendritic cells *in vitro*

**Fig 1:**
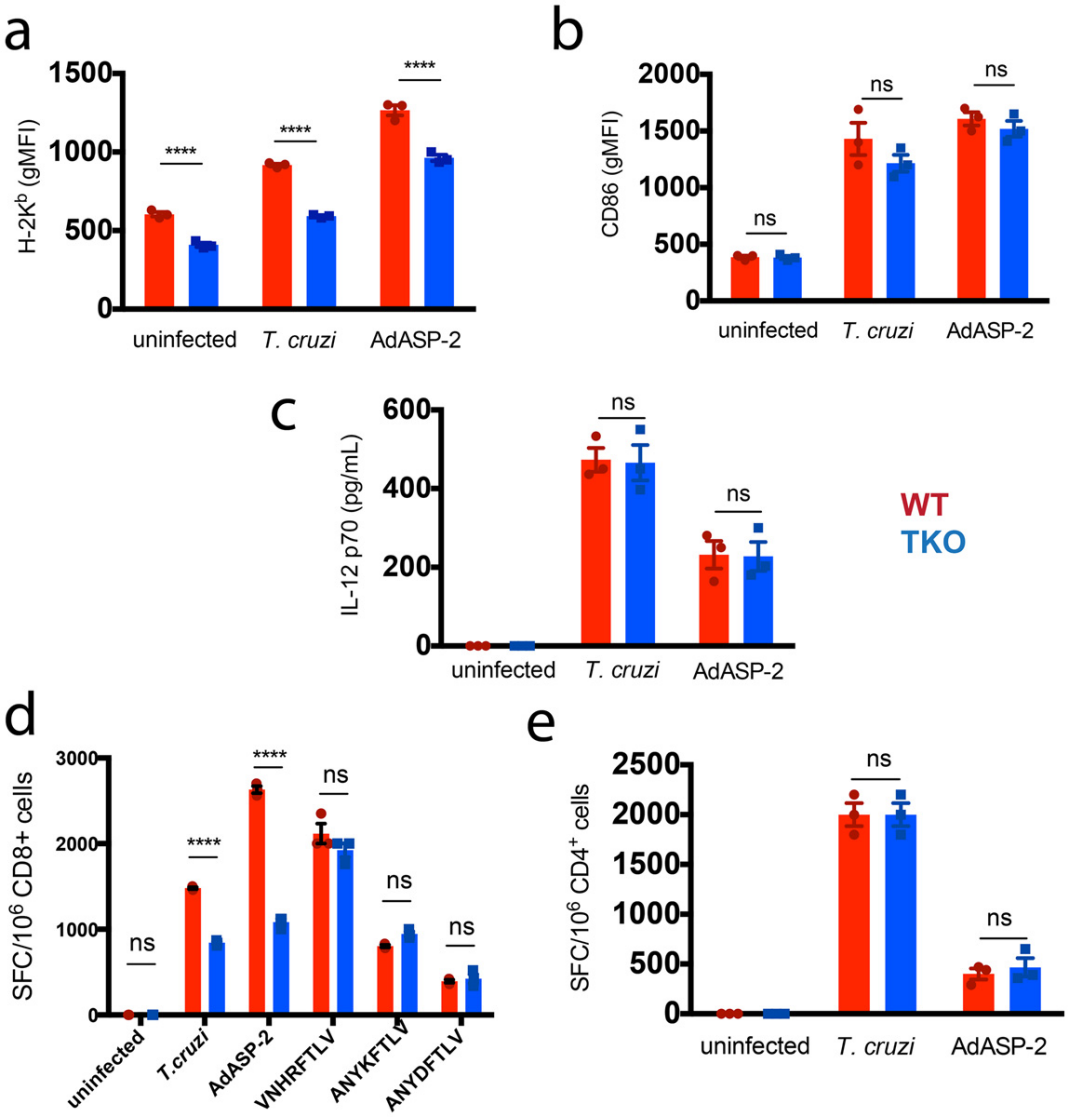
Reduced *in vitro* presentation of MHC class I epitopes from *T*. *cruzi* by immunoproteasome-deficient BMDC. *In vitro*-generated WT (red) or TKO (blue) BMDC were incubated for 24 h with trypomastigotes of *T*. *cruzi* Y strain (m.o.i. = 3), the adenoviral vector AdASP-2 (m.o.i. = 50), or left untreated. (a) Staining of H-2K^b^ and (b) CD86 gated in CD11c^+^ BMDC. (c) IL-12 concentration in culture supernatants obtained after 24 h of cultivation as measured by ELISA. (d) Purified CD8^+^ or (e) CD4^+^ T cells were obtained from the spleens of WT mice infected with *T*. *cruzi* 15 days earlier. The purified T cells were admixed with WT or TKO BMDC and incubated overnight. Where indicated, BMDC were loaded with the peptides VNHRFTLV, ANYKFTLV, or ANYDFLTV, corresponding to H-2K^b^-restricted epitopes from *T*. *cruzi*. The frequency of IFN-γ-producing cells was detected by ELISPOT. SFC: spot-forming cells. Results are shown as individual values and as the mean ± SEM for each group (n = 3). One of two independent experiments is presented. Asterisks indicate that the values observed in TKO mice were significantly lower than those in WT mice (****P<0.0001).

Because dendritic cells constitutively express immunoproteasomes, we examined whether *in vitro*-generated bone marrow-derived dendritic cells (BMDC) from TKO mice differed from WT BMDC in antigen presentation capacity upon exposure to *T*. *cruzi* trypomastigotes or particles of the adenoviral vaccine vector expressing the immunodominant *T*. *cruzi* antigen ASP-2 (AdASP-2) [28]. Upon stimulation with parasites or adenovirus, we observed that WT and TKO BMDCs upregulated the costimulatory marker CD86 equally well *in vitro*, whereas the expression of H-2K^b^ molecules by TKO BMDC was lower than by their WT counterparts (Fig 1a and 1b). In addition, IL-12 p70 concentrations in the culture supernatants were similar between WT BMDC and TKO BMDC (Fig 1c).

BMDC were then co-cultured with purified CD4^+^ or CD8^+^ T cells collected from *T*. *cruzi*-infected mice. Previously, we have confirmed the cytosolic processing of H-2-restricted epitopes from *T*. *cruzi* by incubating these purified T cells *in vitro* with *T*. *cruzi*- or AdASP-2-exposed BMDC deficient in TAP-1 or treated with the proteasome inhibitor epoxomicin (S1 Fig). Consistent with the result showing lower MHC class I expression, TKO BMDC exposed to *T*. *cruzi* or AdASP-2 stimulated significantly fewer IFN-γ-producing CD8^+^ T cells than WT BMDC did, but no difference was observed when BMDC were incubated with synthetic VNHRFTLV peptide corresponding to the immunodominant H-2K^b^-restricted epitope from ASP-2, or the ANYKFTLV and ANYDFTLV peptides that correspond to the respective subdominant epitopes, thus indicating that the reduction in antigen presentation capacity from TKO BMDC is due to the impaired processing of MHC class I-restricted epitopes (p<0.001, Fig 1d). Conversely, we observed that TKO and WT BMDC exposed to *T*. *cruzi* or AdASP-2 were equally able to present MHC class II-restricted epitopes *in* vitro, as measured by their capacity to stimulate similar numbers of IFN-γ-producing CD4^+^ T cells (Fig 1e). When *T*. *cruzi*- or AdASP-2-exposed BMDC were co-cultured with CD4^+^ or CD8^+^ T cells isolated from *naïve* mice, no IFN-γ secretion was detected.

These results thus suggested the contribution of immunoproteasomes for the processing of MHC class I-restricted *T*. *cruzi* epitopes delivered by the parasite itself or by an adenoviral vaccine vector.

### *T*. *cruzi* induces the transcription of immunoproteasome genes in mice and humans

**Fig 2:**
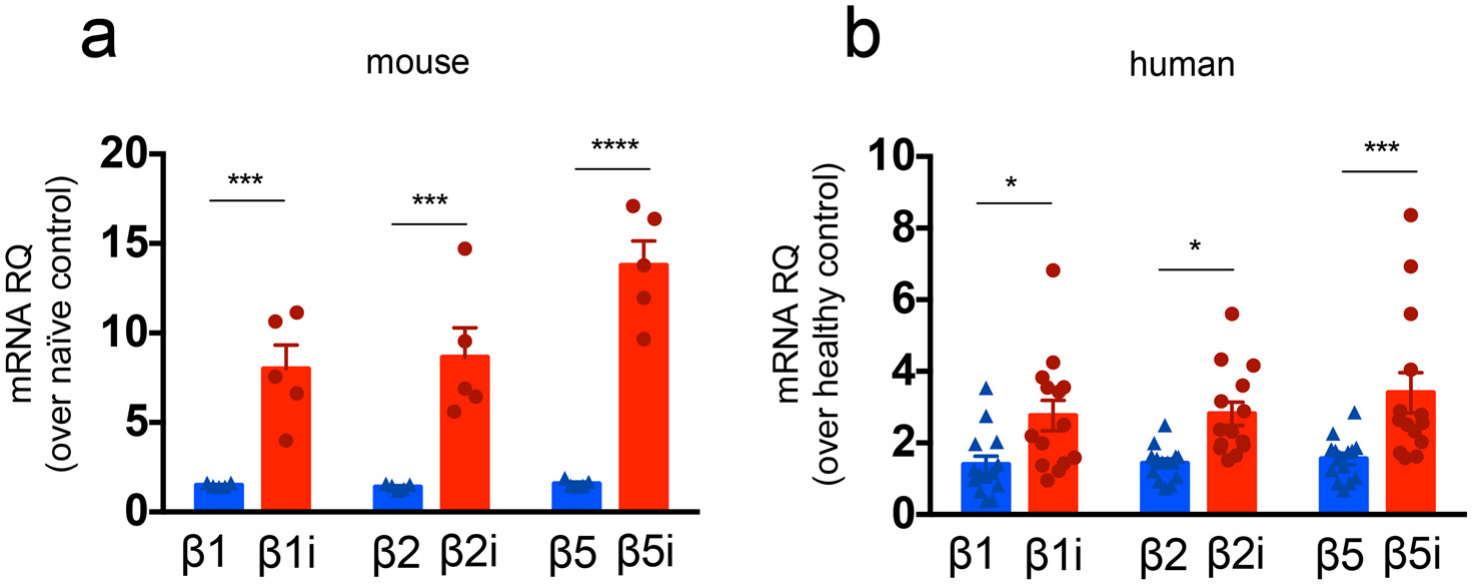
*T*. *cruzi* induces the transcription of immunoproteasome genes in mice and humans. Relative quantification (RQ) of the indicated mRNA levels was measured by real-time PCR in myocardial tissue samples from (a) WT mice infected with *T*. *cruzi* (Y strain) 12 dpi or from (b) chronic chagasic patients with cardiomyopathy as compared to samples obtained from *naïve* mice or healthy subjects, respectively. Results are shown as individual values and as the mean ± SEM for each group. For mouse groups, n = 5, pooled from two independent experiments, and for human samples, n = 14. Asterisks indicate that the values observed for immunoproteasome genes were significantly higher than those for conventional proteasome genes (*P<0.05 ***P<0.001 ****P<0.0001).

To further investigate the participation of immunoproteasomes in the response to *T*. *cruzi* infection *in vivo*, we performed semi-quantitative real-time PCR with primers specific to β1, β2, β5 and the respective surrogate β1i, β2i, and β5i genes using cDNA obtained from heart samples of *naïve* and *T*. *cruzi*-infected mice 12 dpi. Additionally, the corresponding human mRNAs were quantified in heart samples from healthy and chronic chagasic patients with cardiomyopathy. These experiments demonstrated that *in vivo* infection with *T*. *cruzi* induces the transcription of immunoproteasome genes in both mice and humans (Fig 2).

### Impaired immunity of specific CD8^+^ T cells and higher tissue parasitism upon *T*. *cruzi* infection of immunoproteasome-deficient mice

**Fig 3:**
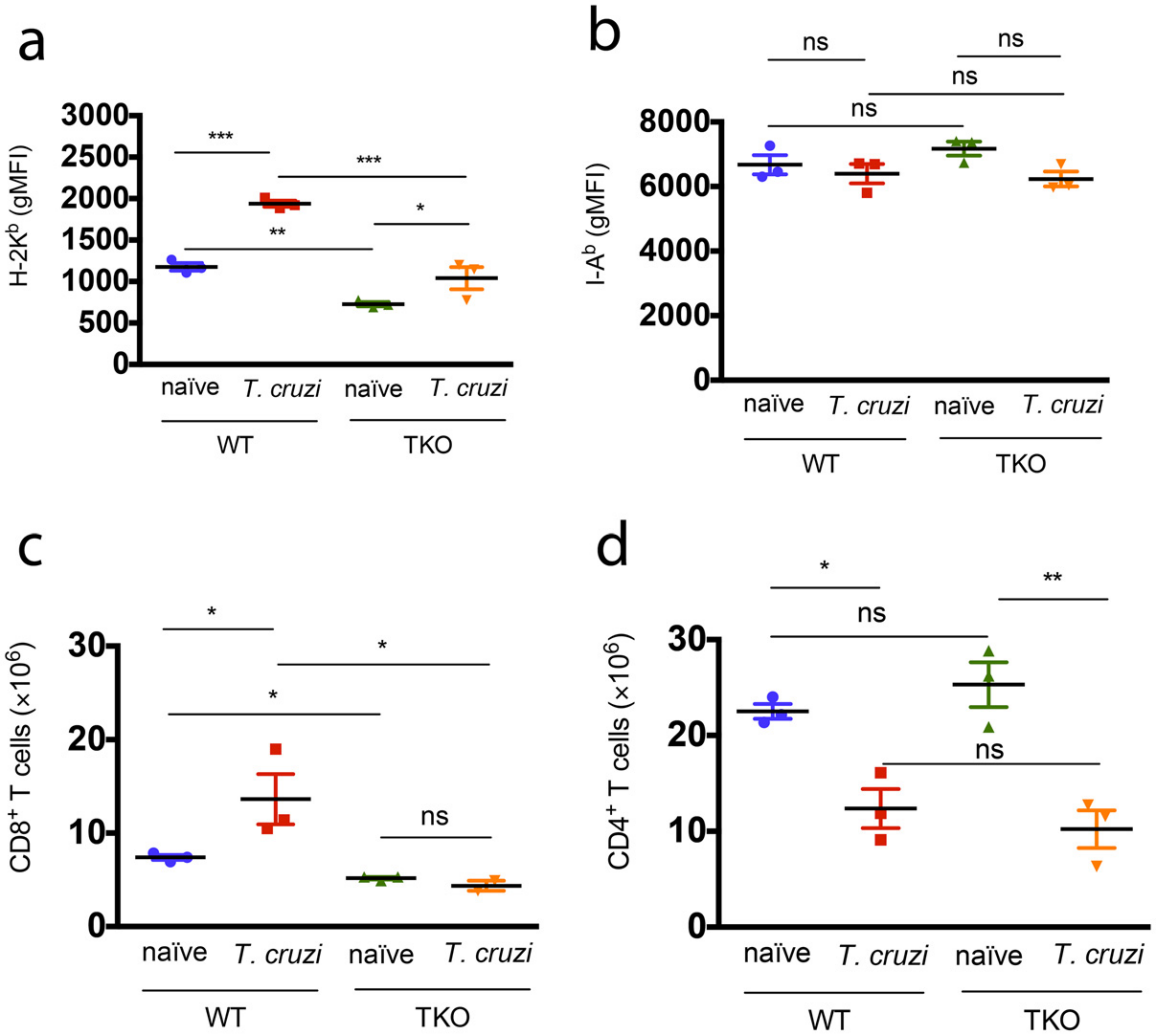
Mice lacking immunoproteasomes have diminished expression of MHC class I molecules and reduced numbers of CD8^+^ T cells. WT and TKO mice were infected *s*.*c*. with 10^4^ *T*. *cruzi* parasites (Y strain) or left uninfected. Twenty days later, spleen cells were collected and stained for surface markers. (a) gMFI (geometric mean of fluorescence intensity) of H-2K^b^ and (b) I-A^b^ stainings are shown after gating in CD11c^+^ I-A^b+^ CD3^-^ CD19^-^ cells. Total numbers of CD8^+^ and CD4^+^ T cells in the spleen of these animals are shown in (c) and (d), respectively. Results are shown as individual values and as the mean ± SEM for each group (n = 3). One representative of at least two independent experiments is shown. Asterisks indicate that the values observed for TKO mice were significantly different than those for WT mice (*P<0.05 **P<0.01 ***P<0.001).

Following experimental infection, we evaluated the expression of MHC molecules by splenic antigen-presenting cells (CD11c^+^ I-A^b+^ CD3^-^ CD19^-^) from *naïve* animals and from mice challenged with *T*. *cruzi* twenty days earlier. As expected, *T*. *cruzi* infection induced the upregulation of MHC class I in both WT and TKO cells (p<0.001 in both cases), suggesting the participation of the conventional immunoproteasome catalytic subunits in the processing of H-2K^b^-restricted epitopes. Nevertheless, the expression of H-2K^b^ molecules on the surface of TKO cells from *naïve* or infected mice was significantly lower than on their counterpart WT cells (p<0.01, Fig 3a), indicating the contribution of immunoproteasomes to the processing of *T*. *cruzi* epitopes. In contrast, the expression of I-A^b^ molecules on the surface of TKO antigen-presenting cells from *naïve* or infected mice was similar to their WT counterparts (Fig 3b). Accordingly, *naïve* and infected TKO mice presented lower numbers of total CD8^+^ T cells in the spleen in comparison to WT animals, whereas no difference in total CD4^+^ T cell numbers was observed between WT and TKO mice (Fig 3c and 3d). When results were expressed in cell frequencies, these differences remained exclusive for the CD8^+^ T cell compartment (S2 Fig).

**Fig 4:**
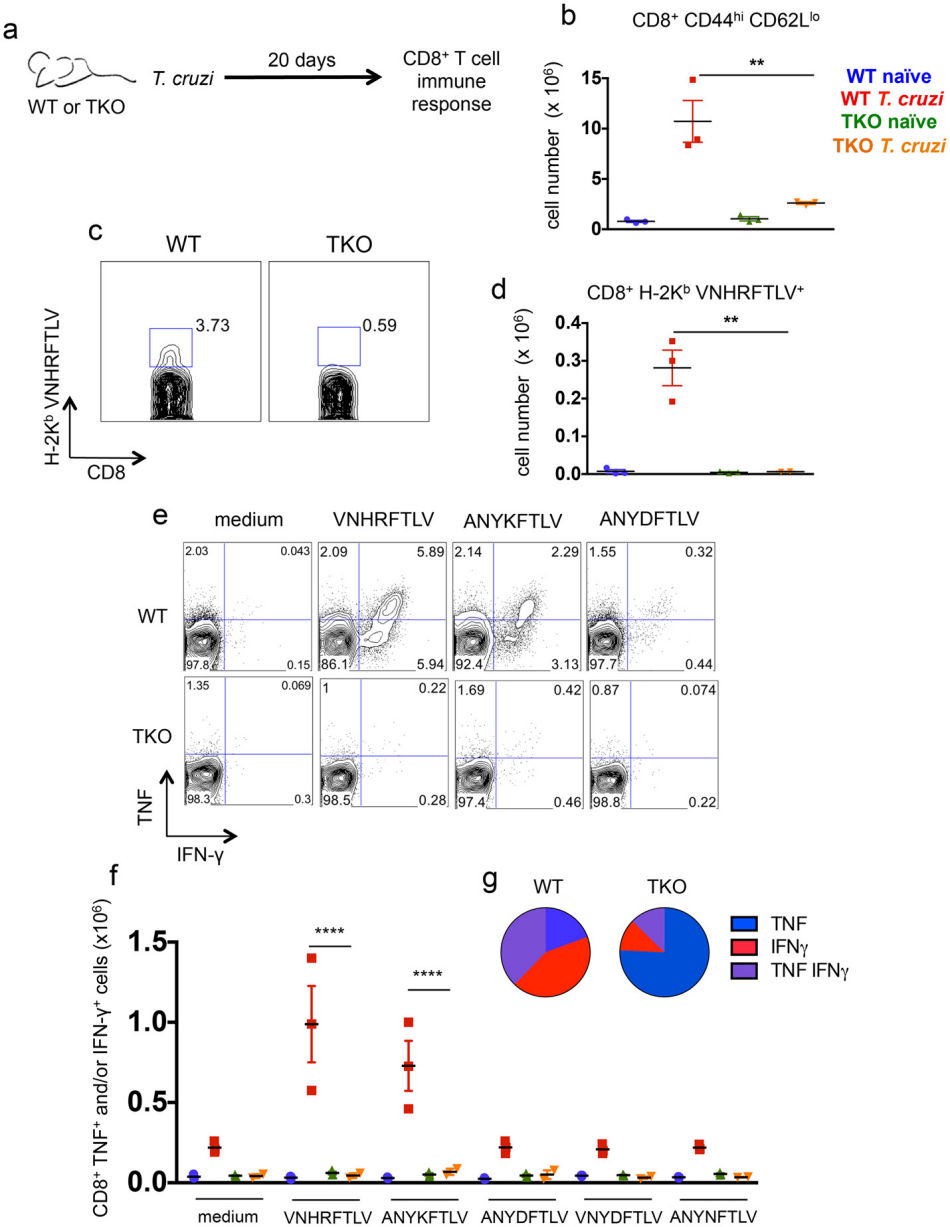
Immunoproteasome-deficient mice present impaired immunity of specific CD8^+^ T cells upon infection with *T*. *cruzi*. (a) Experiment design: WT and TKO mice were infected *s*.*c*. with 10^4^ *T*. *cruzi* parasites (Y strain) or left uninfected. Twenty days later, the response of CD8^+^ T cells was assessed in the spleen. (b) Total numbers of CD8^+^ CD44^high^ CD62L^low^ cells. (c) Representative samples and (d) total numbers of specific CD8^+^ T cells stained with H-2K^b^-VNHRFTLV pentamers. (e) Representative samples and (f) numbers of CD8^+^ splenic cells positively stained with anti-TNF and/or anti-IFN-γ after *ex vivo* restimulation with the indicated peptides corresponding to known or hypothetical *T*. *cruzi* MHC class I-restricted epitopes. (g) Combination of cytokines stained in responder CD8^+^ T cells from spleens of *T*. *cruzi*-infected mice restimulated *ex vivo* with VNHRFTLV peptide. Results are shown as individual values and as the mean ± SEM for each group (n = 3). One representative of two independent experiments is shown. Asterisks indicate that the values observed for TKO mice were significantly lower than those for WT mice (**P<0.01 ****P<0.0001).

The immune response mediated by CD8^+^ T cells was evaluated in detail in WT and TKO mice 20 days after infection with *T*. *cruzi*. The total numbers of splenic CD8_effector_ cells (CD8^+^CD44^high^CD62L^low^) in infected TKO mice were significantly lower than the corresponding numbers in infected WT animals (p<0.01, Fig 4b). Using pentamer staining, we also measured the numbers of CD8^+^ T cells specific for the previously characterized immunodominant, H-2K^b^-restricted, *T*. *cruzi* epitope VNHRFTLV [29, 30]. Again, these numbers were considerably lower in TKO mice than those observed in infected WT mice (p<0.01, Fig 4c and 4d). We further evaluated the function of CD8^+^ T cells through their pattern of IFN-γ/TNF production as assessed by *ex vivo* restimulation of splenocytes with the peptides VNHRFTLV, ANYKFTLV, and ANYDFTLV (corresponding to *T*. *cruzi* H-2K^b^-restricted epitopes) followed by intracellular staining. In presence of the two former peptides, higher numbers of CD8^+^ T cells from infected WT mice produced IFN-γ and/or TNF in comparison to the infected TKO animals, whereas *naïve* mice did not respond regardless of their background (p<0.0001, Fig 4e and 4f). We also stimulated splenocytes with the peptides VNYDFTLV and ANYNFTLV, which have similar sequences to the described H-2K^b^-restricted epitopes from *T*. *cruzi*, in order to test whether they became immunogenic in TKO animals. However, no cytokine response was observed (Fig 4f). These results were similar when expressed in cell frequencies and were further confirmed by estimating the numbers of IFN-γ-producing CD8^+^ T cells by ELISPOT assay after incubation with each peptide (S3 Fig). The specific response to the subdominant epitope ANYDFTLV elicited by *T*. *cruzi* infection was higher in WT mice compared with TKO mice; however, statistical significance was reached only in the ELISPOT assay (S3 Fig). Not only the quantity, but also the quality of specific CD8^+^ T cell cytokine response was altered in TKO animals. Among the cells that produced any cytokine, the frequencies of double-positive (IFN-γ^+^/TNF^+^) or single-positive (IFN-γ^+^) cells after restimulation with VNHRFTLV peptide were higher in infected WT mice compared with cells from infected TKO mice (p<0.01, Fig 4g), whereas TNF single-positive cells prevailed among CD8^+^ T cells from TKO mice (Fig 4g).

We also compared the frequencies of splenic CD4_effector_ (CD4^+^CD44^high^CD62L^low^) cells in mice infected 20 days earlier, and we also observed that upon infection, CD4^+^ T cells expressing IFN-γ and/or TNF can be detected without *ex vivo* restimulation, as previously reported [31]. No difference was observed in the effector phenotype or function of CD4^+^ T cells between WT and TKO mice infected with *T*. *cruzi* (S4 Fig).

Based on the experiments above, we concluded that following infection with *T*. *cruzi*, the generation of specific CD8^+^ T cells was severely impaired and profile of cytokine production altered in TKO mice.

**Fig 5:**
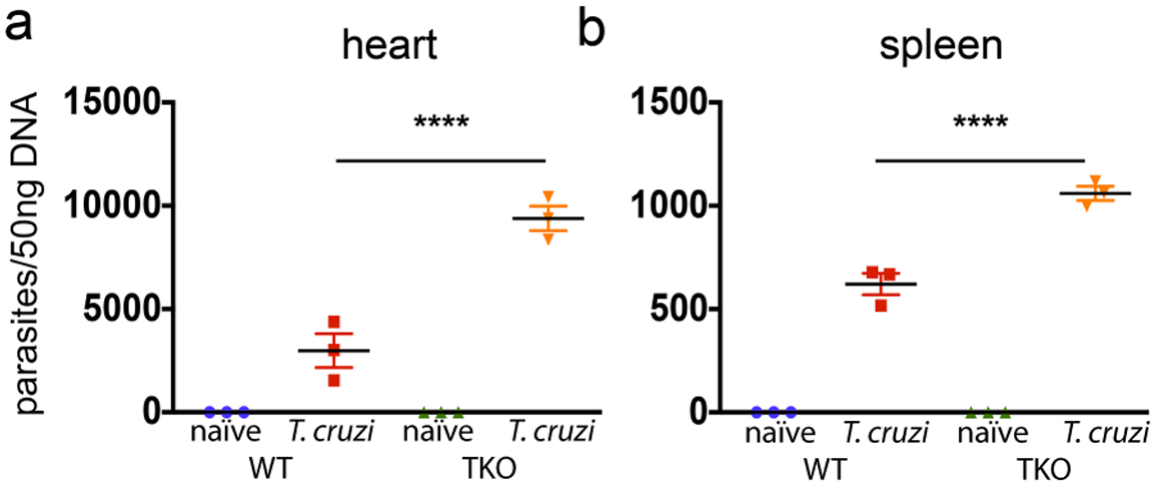
Mice devoid of immunoproteasomes present higher tissue parasitism upon infection with *T*. *cruzi*. WT and TKO mice were infected *s*.*c*. with 10^4^ *T*. *cruzi* parasites (Y strain) or left uninfected. Twenty days later, their (a) hearts and (b) spleens were collected, and the number of parasites/50 ng genomic DNA was measured by real-time PCR. Results are shown as individual values and as the mean ± SEM for each group (n = 3). One representative of two independent experiments is shown. Asterisks indicate that the values observed for TKO mice were significantly higher than those for WT mice (****P<0.0001).

To test whether the impaired immunity of CD8^+^ T cells in TKO mice correlates with reduced resistance to infection with *T*. *cruzi*, we further estimated the amount of parasite DNA in infected WT and TKO mice. As shown in Fig 5, in the heart and spleen, the quantity of *T*. *cruzi* DNA was significantly higher in TKO mice as compared with WT animals (p<0.0001).

### Failure of genetic vaccination in mice devoid of immunoproteasomes

Given that CD8^+^ T cells are critical for immunity against *T*. *cruzi* infection, we developed an immunization regimen that successfully vaccinates highly susceptible mice against systemic lethal infection [32–34]. For that purpose, we used recombinant plasmid DNA for priming and human replication-defective recombinant adenovirus type 5 for boost, both vectors expressing ASP-2 of *T*. *cruzi*. This vaccination protocol elicited a long-lived protective immune response mediated by CD8^+^ effector and effector memory T cells [35, 36].

**Fig 6:**
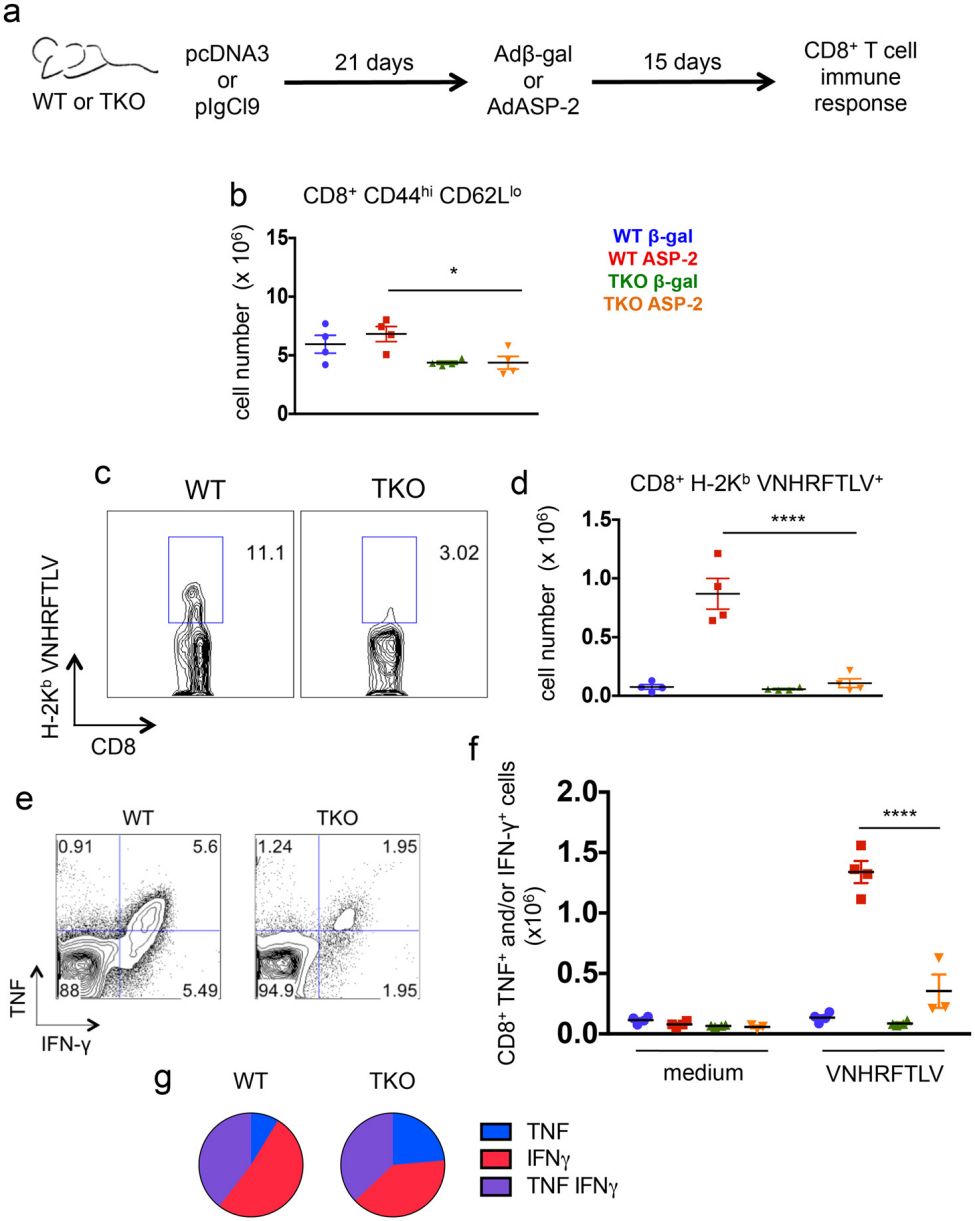
Immunoproteasome-deficient mice present impaired immunity of specific CD8^+^ T cells upon genetic vaccination against *T*. *cruzi*. (a) Experiment design: WT and TKO mice were primed with empty plasmid DNA (pcDNA3) or a vector expressing ASP-2 (pIgCl9) and boosted after 21 days with adenovirus 5 expressing beta-galactosidase (Adβ-gal) or ASP-2 (AdASP-2), respectively. Fifteen days later, the response of CD8^+^ T cells was assessed in the spleen. (b) Total numbers of CD8^+^ CD44^high^ CD62L^low^ cells. (c) Representative samples and (d) total numbers of specific CD8^+^ T cells stained with H-2K^b^-VNHRFTLV pentamers. (e) Representative samples and (f) numbers of CD8^+^ splenic cells positively stained for TNF and/or IFN-γ after *ex vivo* restimulation with the peptide VNHRFTLV corresponding to the immunodominant *T*. *cruzi* MHC class I-restricted epitope from ASP-2. (g) Combination of cytokines stained in responder CD8^+^ T cells from spleens of ASP-2-vaccinated mice restimulated *ex vivo* with VNHRFTLV peptide. Results are shown as individual values and as the mean ± SEM for each group (n = 4). One representative of two independent experiments is shown. Asterisks indicate that the values observed for TKO mice were significantly lower than those for WT mice (*P<0.05 ****P<0.0001).

When we compared the response of CD8^+^ T cells, we found significantly lower numbers of CD8_effector_ cells in ASP-2-vaccinated TKO mice than in ASP-2-vaccinated WT animals (p<0.05, Fig 6b). As in the case of *T*. *cruzi*-infected mice, the numbers of cells specific for the VNHRFTLV epitope among the splenic CD8^+^ T cells of ASP-2-vaccinated TKO mice were significantly lower than those observed in ASP-2-vaccinated WT mice (p<0.0001, Fig 6c and 6d). In addition, the numbers of specific CD8^+^ T cells producing IFN-γ and/or TNF upon *ex vivo* restimulation with the peptide VNHRFTLV were also lower in the population of splenic CD8^+^ T cells from ASP-2-vaccinated TKO mice compared with cells from ASP-2-vaccinated WT mice (p<0.0001, Fig 6e and 6f). The quality of the immune response of the CD8^+^ T cells from ASP-2-vaccinated WT mice in comparison to TKO animals was not as different as observed in *T*. *cruzi*-infected mice, and IFN-γ single-positive or IFN-γ/TNF double-positive cells predominated in both WT and TKO animals (Fig 6g). When expressed in cell frequencies, or when the cytokine response was assessed by ELISPOT, similar differences between WT and TKO mice were found (S5 Fig). Moreover, the response of CD4^+^ T cells to genetic vaccination with *Asp-2* was similar between WT and TKO animals, as measured by *in vivo* incorporation of BrdU in CD44^high^ cells or intracelllular staining of IFN-γ after *ex-vivo* restimulation with AdASP-2-infected cells (S6 Fig). Overall, we concluded that following genetic immunization or infection with *T*. *cruzi*, TKO mice were severely impaired in the generation of CD8^+^ T cell-mediated immune responses to the VNHRFTLV epitope.

**Fig 7:**
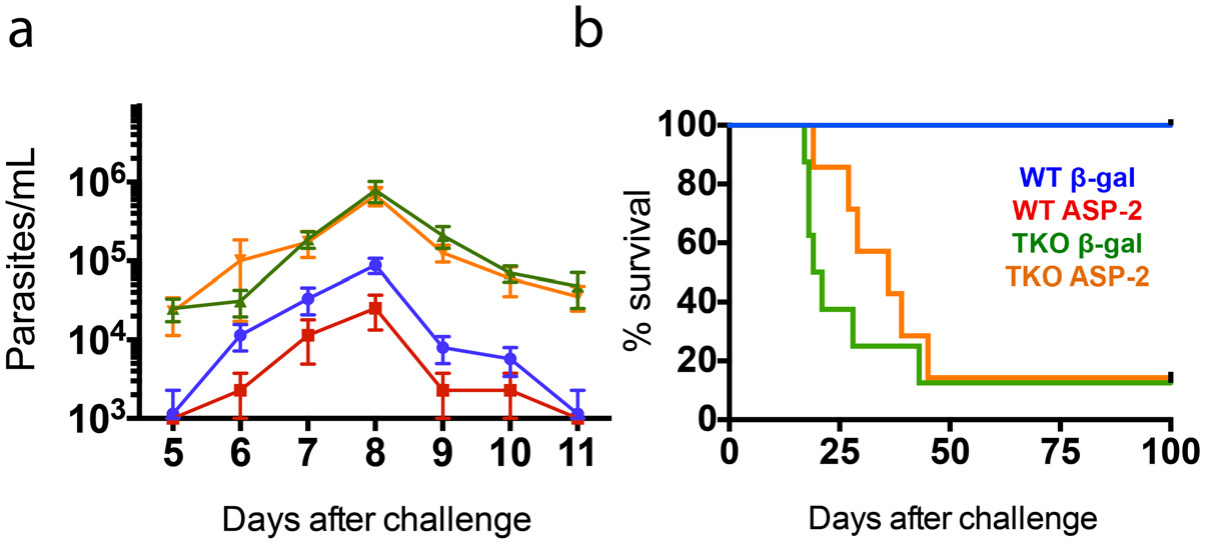
Failure of genetic vaccination-induced protection against *T*. *cruzi* in mice lacking immunoproteasomes. WT and TKO mice (n = 7–8) were primed with empty plasmid DNA (pcDNA3) or a plasmid vector expressing ASP-2 (pIgCl9) and boosted after 21 days with adenovirus 5 expressing beta-galactosidase (Adβ-gal) or ASP-2 (AdASP-2), respectively. Fifteen days later, the mice were challenged *s*.*c*. with 10^4^ *T*. *cruzi* parasites (Y strain). (a) Parasitemia and (b) Kaplan-Meier survival curves for each mouse group are presented. Parasitemia is expressed as the mean ± SEM. The values of peak parasitemia (day 8) were log transformed and compared by one-way ANOVA followed by Tukey’s HSD test, which showed that i) ASP-2-immunized WT mice displayed levels of parasitemia significantly lower than those of the other mouse groups (p<0.01 in all cases), ii) the parasitemia values of TKO mice (vaccinated or control) were higher than those of WT mice (p<0.01 in all cases), and iii) no difference between vaccinated and control mice was detected among TKO animals. Comparison of the survival curves using the log-rank (Mantel-Cox) test indicated that i) vaccinated and control WT mice survived significantly longer (p<0.001) than TKO mice did, and ii) although vaccinated TKO mice survived slightly longer than control TKO mice did, no statistical significance was reached. Data was pooled from three independent experiments (n = 7–8).

The ultimate aim of our study was to determine whether immunoproteasomes are important for resistance against infection with *T*. *cruzi*. Because MHC I-restricted CD8^+^ T cells have been described as important for protective immunity in both naïve and vaccinated mice, as estimated based on parasitemia and mouse survival, we expected that TKO mice would be more susceptible to infection than their WT counterparts. Accordingly, we observed that after challenge, TKO mice vaccinated with the βgal unrelated control or with ASP-2 presented levels of parasitemia that were about one order of magnitude higher than those in WT mice vaccinated with the βgal control (p<0.01, Fig 7a). Notably, the parasitemia detected in TKO mice previously immunized with ASP-2 was indistinguishable from that observed among TKO mice that had received the unrelated βgal-expressing vector. In contrast, and as previously described [32], the parasitemia observed after challenge of ASP-2-vaccinated WT mice was significantly lower than that measured among βgal-vaccinated control animals (p<0.01, Fig 7a). Not only did TKO mice have higher parasitemia, but most of them also succumbed to an otherwise non-lethal infection. A total of 87.5% of TKO mice vaccinated with the βgal control succumbed before 45 days after infection. ASP-2-vaccinated TKO mice survived slightly longer, but still, 85.7% of the mice died before day 50 after challenge (Fig 7b). The increase in survival was statistically significant in the groups of WT mice compared with TKO mice (p<0.001). These results support the association between the decrease in the frequency of specific splenic CD8^+^ T cells and the limited parasite control in TKO mice.

Because different strains of *T*. *cruzi* may present distinct patterns of infectivity in mice, we also tested whether TKO mice were more susceptible to infection with parasites of the CL strain. Similar to the case of mice infected with parasites of the Y strain, we observed that TKO mice were highly susceptible to infection and unable to control their parasitemia, succumbing before 25 days following infection with an otherwise non-lethal challenge (S7 Fig).

**Fig 8:**
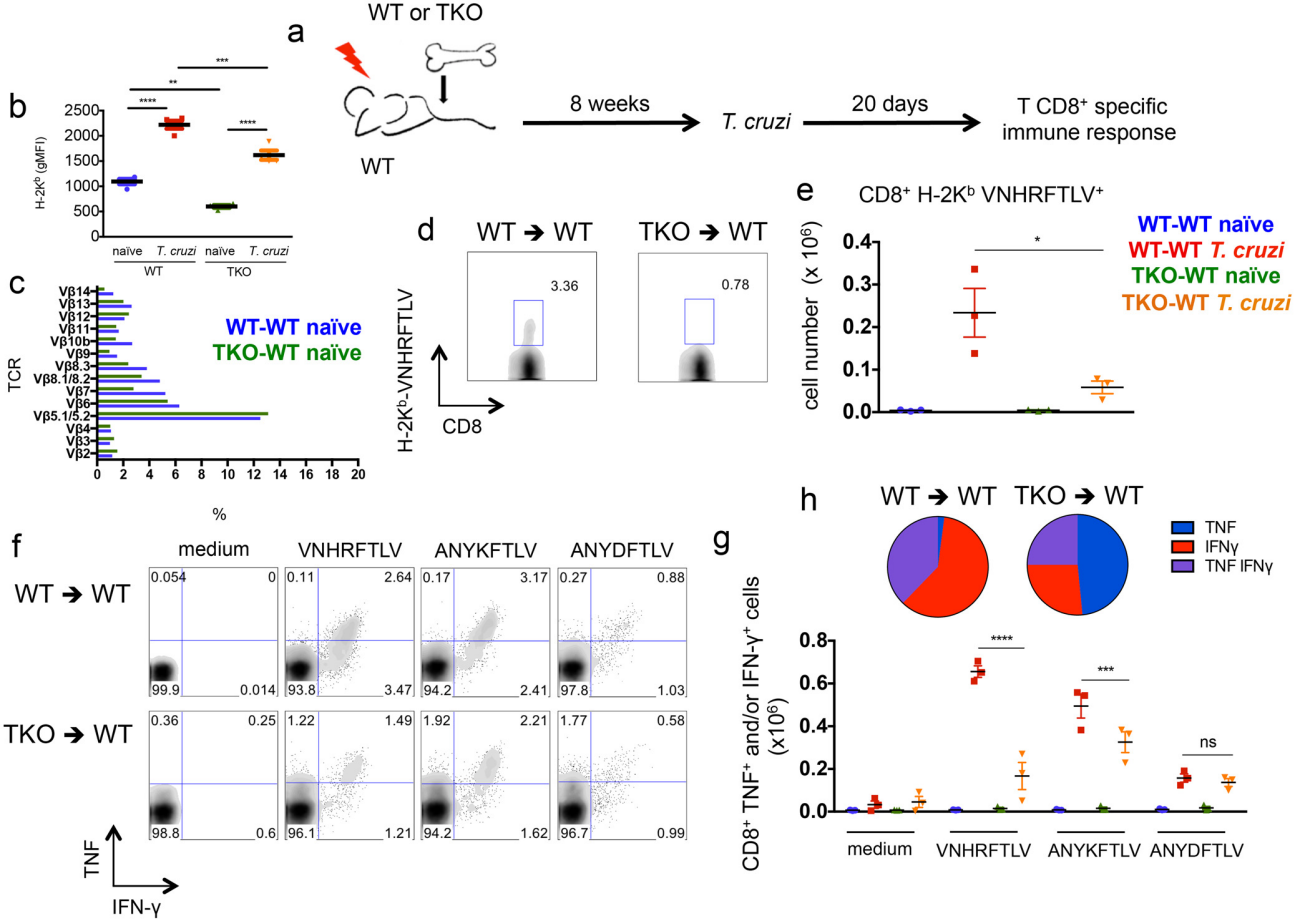
Impaired immunity of specific CD8^+^ T cells upon infection with *T*. *cruzi* of WT mice reconstituted with immunoproteasome-deficient bone marrow. (a) Experiment design: WT mice were irradiated and reconstituted with WT (WT-WT) or TKO (TKO-WT) bone marrow. After 8 weeks, the chimeric animals were infected *s*.*c*. with 10^4^ *T*. *cruzi* parasites or left uninfected. Twenty days later, the response of CD8^+^ T cells was assessed in the spleen. (b) gMFI (geometric mean of fluorescence intensity) of H-2K^b^ staining of CD11c^+^ splenic cells from WT-WT and TKO-WT chimeras. (c) Staining of TCR Vβ chains gated in CD8+ T cells from *naïve* WT-WT and TKO-WT chimeras. (d) Representative samples and (e) total numbers of specific CD8^+^ T cells stained with H-2K^b^-VNHRFTLV pentamers. (f) Representative samples and (g) total numbers of CD8^+^ splenic cells positively stained with anti-TNF and/or anti-IFN-γ after *ex vivo* restimulation with the indicated peptides corresponding to known or hypothetical *T*. *cruzi* MHC class I-restricted epitopes. (h) Combination of cytokines stained in responder CD8^+^ T cells from spleens of *T*. *cruzi*-infected mice restimulated *ex vivo* with VNHRFTLV peptide. Results are shown as individual values and as the mean ± SEM for each group (n = 3). One representative of two independent experiments is shown. Asterisks indicate that the values observed for TKO mice were significantly lower than those for WT mice (*P<0.05 **P<0.01 ***P<0.001 ****P<0.0001).

An altered CD8^+^ T cell repertoire has been previously described in TKO mice in comparison to WT animals [26]. This was explained by a different repertoire of immunogenic peptides presented by thymic epithelial cells from TKO mice. To test whether a difference in the T cell repertoire accounted for most of the defective immune response observed in TKO mice, we generated bone marrow chimeras. Irradiated WT mice were reconstituted with bone marrow from either WT (WT-WT) or TKO (TKO-WT) animals and after 8 weeks these mice were infected with *T*. *cruzi*. After 20 days of infection, the response of CD8^+^ T cells was assessed (Fig 8a). In this set up, bone marrow-derived antigen presenting cells lack immunoproteasomes in TKO-WT chimeras, as indicated by the lower expression of H-2K^b^ molecules on CD11c^+^ splenic cells from these animals in comparison to WT-WT mice (Fig 8b). Conversely, epithelial thymic stromal cells are WT in both WT-WT and TKO-WT chimeras, thus allowing similar selection of CD8^+^ T cells. Similar CD8^+^ T cell repertoire between WT-WT and TKO-WT chimeras was inferred by the indistinguishable staining of CD8^+^ T cells with TCR Vβ antibodies panel (Fig 8c). Upon *T*. *cruzi* infection, the chimeric TKO-WT animals presented lower numbers of VNHRFTLV-specific CD8^+^ T cells stained with pentamers (p<0.05, Fig 8d and 8e) and lower numbers of cytokine-producing CD8^+^ T cells specific to VNHRFTLV and ANYKFTLV epitopes (p<0.001, Fig 8f and 8g), although the response to the subdominant epitope ANYDFTLV was comparable between WT-WT and TKO-WT chimeras (Fig 8f and 8g). Once again, the frequency of double-positive (IFN-γ^+^/TNF^+^) or single-positive (IFN-γ^+^) CD8^+^ T cells after restimulation with VNHRFTLV peptide was higher in infected WT-WT chimeric mice compared with cells from infected TKO-WT mice (p<0.01, Fig 8h).

**Fig 9:**
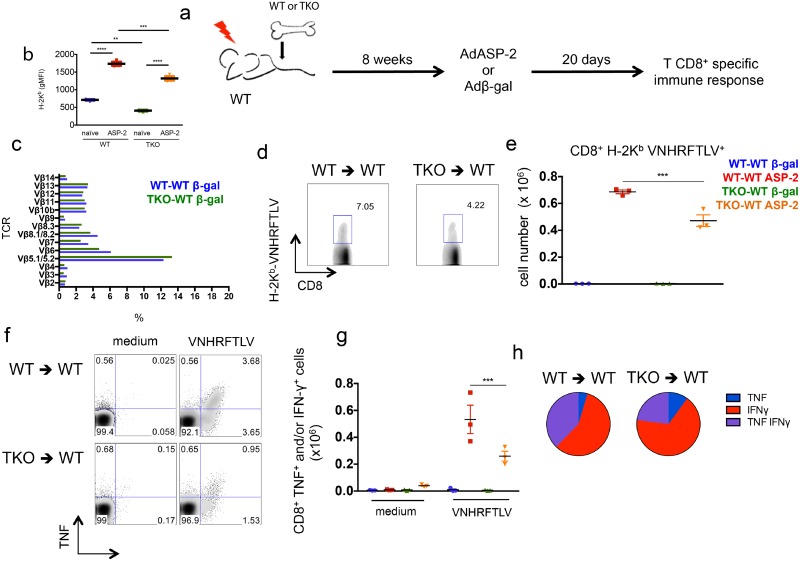
Impaired immunity of specific CD8^+^ T cells upon genetic vaccination of WT mice reconstituted with immunoproteasome-deficient bone marrow. (a) Experiment design: WT mice were irradiated and reconstituted with WT (WT-WT) or TKO (TKO-WT) bone marrow. After 8 weeks, chimeric mice were vaccinated with adenovirus 5 expressing beta-galactosidase (Adβ-gal) or ASP-2 (AdASP-2). Twenty days later, the response of CD8^+^ T cells was assessed in the spleen. (b) gMFI (geometric mean of fluorescence intensity) of H-2K^b^ staining of CD11c^+^ splenic cells from WT-WT and TKO-WT chimeras. (c) Staining of TCR Vβ chains gated in CD8+ T cells from WT-WT and TKO-WT chimeras genetically immunized with Adβ-gal. (d) Representative samples and (e) total numbers of specific CD8^+^ T cells stained with H-2K^b^-VNHRFTLV pentamers. (f) Representative samples and (g) total numbers of CD8^+^ splenic cells positively stained with anti-TNF and/or anti-IFN-γ after *ex vivo* restimulation with the peptide VNHRFTLV corresponding to the immunodominant MHC class I-restricted epitope from ASP-2. (h) Combination of cytokines stained in responder CD8^+^ T cells from spleens of AdASP-2-vaccinated mice restimulated *ex vivo* with VNHRFTLV peptide. Results are shown as individual values and as the mean ± SEM for each group (n = 3). One representative of two independent experiments is shown. Asterisks indicate that the values observed for TKO mice were significantly lower than those for WT mice (**P<0.01 ***P<0.001 ****P<0.0001).

Moreover, WT-WT and TKO-WT chimeras were vaccinated with AdASP-2 and the specific CD8^+^ T cell response was evaluated after 20 days (Fig 9a). Again, splenic CD11c^+^ cells from TKO-WT mice presented lower expression of H-2K^b^ (Fig 9b), whereas the staining of CD8^+^ T cells with TCR Vβ antibodies panel was similar between TKO-WT and WT-WT animals (Fig 9c). Despite homogenous repertoire of CD8^+^ T cells, the number and cytokine effector function of VNHRFTLV-specific CD8^+^ T cells was significantly reduced in AdASP-2-vaccinated TKO-WT mice in comparison to AdASP-2-vaccinated WT-WT mice (Fig 9d–9h).

Because even WT-WT chimeric mice became highly susceptible to *T*. *cruzi* challenge, succumbing to infection from 22 days after challenge, comparisons between WT-WT and TKO-WT animals in terms of resistance to infection and vaccine-induced protection were not possible. Nonetheless, these experiments sustain the notion that the impaired immunity of CD8^+^ T cells in *T*. *cruzi*-infected or genetically vaccinated animals lacking all immunoproteasome genes could not be solely attributed to an altered T cell repertoire.

## Discussion

The recognition by CD8^+^ T lymphocytes of antigens displayed on the context of MHC class I molecules is a fundamental requirement for the control of tumors and intracellular infections by viruses, fungi, bacteria and parasites. Proteasomes are of paramount importance in the processing of antigens in the MHC class I pathway. However, the specific contribution of alternative proteasome catalytic subunits β1i, β2i and β5i (as opposed to the canonical active sites) to the generation of MHC class I-restricted epitope repertoire was thought to be, at most, incremental until the recent development of a mouse model simultaneously devoid of β1i, β2i and β5i subunits (TKO mice) [26]. In that study, major differences in the quantity and quality of epitopes recognized by CD8^+^ T cells was reported, suggesting that models where only one or two subunits are targeted might underestimate the relevance of immunoproteasomes in antigen processing and presentation. Although it is reasonable to assume that immunoproteasome’s role in epitope abundance and specificity may lead to altered resistance to a pathogen, supportive data to this assumption remain relatively scarce. Here we explored the TKO mouse model to further investigate the role of immunoproteasomes in the development of protective immunity to a pathogen. *Trypanosoma cruzi*, a neglected human parasite, was of didactical use as a model, since protective immunity against it is highly dependent on CD8^+^ T cell function and IFN-γ (the major inducer of immunoproteasomes). The TKO mice exhibited a drastically reduced response of CD8^+^ T cells specific for immunodominant and subdominant epitopes after *T*. *cruzi* infection, whereas the response of CD4^+^ T cells was unaltered. Surprisingly, natural resistance to infection in the absence of immunoproteasomes was seriously compromised and even more unexpectedly the protection induced by genetic vaccination was completely abolished, indicating that the immunoproteasome catalytic subunits, rather than the conventional proteolytical sites, are essential for the processing of *T*. *cruzi* epitopes related to protective immunity generated during infection or vaccination. Our observations from chimeric mice are consistent with the hypothesis that the defect in TKO mice is the inability of antigen presenting cells to process protective epitopes to CD8^+^ T cells, rather than the variation of naïve CD8^+^ T cell repertoire. Also, we were unable to detect any shift in the immunodominance pattern of TKO mice relatively to WT counterparts.

We have previously reported that the induction of CD8^+^ T cell responses to *T*. *cruzi* is remarkably delayed and suboptimal, with most cells expressing high levels of the proapoptotic marker CD95 [16]. Conversely, genetic vaccination with adenoviral vectors induces a rapid, expanded, polyfunctional and highly viable CD8^+^ T cell response that differentiates into a long-lasting memory pool, as reported for *T*. *cruzi* as well as other pathogens [33–36]. Here, we report that the function of *T*. *cruzi*-specific CD8^+^ T cell responses was altered in the absence of immunoproteasomes, as indicated by the higher frequency of TNF single-positive cells in TKO animals as opposed to a prevailing compartment of IFN-γ single-positive or IFN-γ and TNF double-positive cells in immunoproteasome-sufficient mice. This difference, however, was more pronounced during infection than upon genetic vaccination with *Asp-2*. It is acknowledged that IFN-γ is of major importance for control of *T*. *cruzi* infection and its induction is tightly regulated, whereas TNF, expressed throughout the course of infection, is of secondary importance to parasite control [13, 27, 37]. Our results may suggest that in our model the threshold of antigen presentation required to induce TNF production by CD8^+^ T cells might be lower than the levels of stimulation necessary to induce IFN-γ. Consistently, the data presented here reinforce the notion that AdASP-2 is remarkably more potent than *T*. *cruzi* at inducing CD8^+^ T cell responses, since even in the absence of immunoproteasomes the viral vector was able to stimulate the production of IFN-γ-secreting cells. Therefore, even in the absence of immunoproteasomes, the antigen pool generated by the adenoviral vaccine could be cleaved by conventional proteasomes without severely compromising the induction of polyfuncional CD8^+^ T cells (yet lower number of cells are induced), in contrast to the observations from experimental infection. In fact, we have previously observed that CD8^+^ T cells specific to subdominant epitopes from *T*. *cruzi* can be induced by adenoviral vaccination, but not by protozoan infection, in line with the idea that the provision of epitopes is enhanced during genetic vaccination [33].

Presumably, the lower expression of MHC class I in TKO mice is inextricably linked to a defect in generating peptides that bind to H-2 molecules. We found that upon infection with *T*. *cruzi* or adenovirus expressing ASP-2, dendritic cells from TKO background were highly impaired at presenting antigen to WT CD8^+^ T lymphocytes explanted from either infected or vaccinated mice. These results contrasted with the fact that WT and TKO BMDC loaded with VNHRFTLV, ANYKFTLV, or ANYDFTLV peptides were equally able to stimulate *T*. *cruzi*-specific CD8^+^ T cells *in vitro*. These results suggest that the defects in antigen presentation capacity of TKO cells are due to the compromised processing of epitopes. These findings are consistent with a previous study indicating that in absence of immunoproteasomes the presentation of protein antigens that need to be cleaved is compromised, whereas no defect is observed when the processed epitope is directly expressed by a minigene [26]. Nonetheless, dendritic cells lacking immunoproteasomes still upregulate MHC class I molecules and present epitopes recognized by CD8+ T cells upon infection with *T*. *cruzi* or genetic vaccination with *Asp-2*, an effect most likely attributed to the processing of antigen by the canonical catalytic subunits of the proteasome.

In addition to the poor antigen-presenting function of TKO cells, the immunodominance of epitopes could have been shifted due to the absence of immunoproteasomes, as previously described [38–40]. Although we only tested peptides representing 3 known epitopes and 2 other hypothetical ones, we could not find relevant changes in the immunodominance pattern. Independently of their specificities or hierarchies, the CD8^+^ T cells activated in TKO mice were always reduced in comparison to WT counterparts.

An altered CD8^+^ T cell repertoire has been previously described in TKO mice in comparison to WT animals [26]. Hence, distinct naïve T cell repertoires could also explain the discrepancies in CD8^+^ T cell response between TKO and WT mice [26, 41]. It is assumed that this difference in the repertoire of CD8^+^ T cells from WT and TKO mice is due to MHC I-mediated presentation of a different repertoire of peptides by epithelial cells from thymus during T cell development. To address this issue we performed experiments using chimeras, where recipient WT mice received bone marrows from either WT or TKO mice. The results obtained from these experiments suggest that it is unlikely that a difference in the T cell repertoire accounted for most of the limited immune response observed in TKO mice. In the same direction, the transfer into TKO mice of WT CD8^+^ T cells or of the P14 transgenic CD8^+^ T cell clone specific to GP33 epitope was unable to restore the capacity of TKO animals to optimally respond to LCMV infection [26].

Not only did TKO mice have diminished CD8^+^ T cell immune responses to parasite-derived epitopes, but they were also no longer resistant to infection with *T*. *cruzi*. It is reasonable to hypothesize that the susceptibility to infection and the weak CD8^+^ T cell-mediated immunity are linked because in the mouse models that we used (*naïve* and vaccinated), CD8^+^ T cells have been described as being critically involved in the control of parasitemia and in survival [30, 32]. Nevertheless, in addition to the low numbers of specific CD8^+^ T cells, other factor(s) may also account for the extreme susceptibility that we observed. For instance, a second likely source of reduced resistance may stem from the effector phase of the immune response. During *T*. *cruzi* infection, large amounts of IFN-γ are produced and can be detected in the serum [42]. As immunoproteasomes become the dominant form of proteasomes in IFN-γ-activated cells, their absence may drastically reduce the antigen presentation capacity of target cells infected with *T*. *cruzi*. This aspect may further contribute to the impaired efficacy of the few CD8^+^ T cells generated during the priming phase.

It is worth mentioning that the susceptibility of TKO mice occurred in parallel to undetectable changes in presentation of MHC II-restricted epitopes to CD4^+^ T cells and in the generation of CD4_effector_ cells during infection. These findings are also in agreement with previous observations that CD4^+^ T cell-mediated immune responses were similar in TKO and WT mice in different infection models [26].

The susceptibility of TKO mice may also be linked to a larger inflammatory reaction and to more severe myocardial tissue damage. Recently, immunoproteasomes have been described as protecting the heart from excessive inflammatory tissue damage due to acute coxsackievirus B3 (CVB3)-induced myocarditis [43]. Because *T*. *cruzi* may infect myocardial cells, whether a similar event is associated with susceptibility to infection should be further investigated. This topic may not be as important in the model of infection used in most of our studies, considering that infection with parasites of the Y strain targets the spleen as well as the heart tissues [44]. However, other *T*. *cruzi* strains, such as the CL or the Colombian strain, cause severe acute heart muscle injury and deserve further investigation [45].

Previous studies using mice genetically deficient for a single immunoproteasome subunit (LMP7) also described poor induction of CD8^+^ T cells, but not CD4^+^ T cells, specific for antigens of *Toxoplasma gondii*. As in our system, these mice were susceptible to an otherwise non-lethal infection [46]. These results further corroborate the interpretation that immunoproteasomes are critical for the generation of intracellular parasite epitopes used for CD8^+^ T cell activation. Additionally, in the absence of immunoproteasomes, mouse survival after infection with *T*. *cruzi* or *T*. *gondii* is compromised, pointing to the immunoproteasomes as key mediators of resistance to intracellular parasite infections.

In other words, these protozoan infection models were instrumental at evidencing a specific role of immunoproteasomes for the generation of critical epitopes required for protective immunity.

By employing adenoviral vectors, our group developed genetic vaccination strategies against *T*. *cruzi*. The immunization regimens confer protection to wild-type mice of different strains, including the C57BL/6, BALB/c and A/Sn [16, 28]. From these, the A/Sn mice are the most susceptible and succumb to infection in less than 30 days, even when challenged with as low as 150 parasites of *T*. *cruzi* Y strain. Conversely, A/Sn mice survive *T*. *cruzi* infection if immunized with AdASP-2 even on the same day of challenge [16]. At least in our animal facility, the C57BL/6 mouse lineage is more resistant to *T*. *cruzi* infection and survives challenge with high doses (10,000) of blood forms of *T*. *cruzi*. Therefore, the C57BL/6 mouse serves as a model to study different aspects of the CD8^+^ T cell response related to resistance. Here, we observed that mice devoid of immunoproteasomes in a C57BL/6 background are highly susceptible to *T*. *cruzi* challenge. Unexpectedly, vaccination of these mice with AdASP-2 does not have any effect in protecting the mice upon challenge, whereas in normal C57BL/6 mice the parasitemia is reduced in about one order of magnitude. These results thus indicate that the efficacy of our vaccination regimen is extremely dependent on the expression of β1i, β2i and β5i subunits of the immunoproteasome, rather than the conventional proteolytical sites. In accordance with this finding, a recent clinical trial aimed at testing the efficacy of the RTS,S vaccine against malaria identified the upregulation of immunoproteasome genes among protected individuals after challenge [47]. Altogether, these results may point to the induction of immunoproteasome genes as pivotal targets to be considered in the design of successful vaccination strategies aimed at inducing CD8^+^ T cells.

In conclusion, we report that immunoproteasomes, rather than canonical proteasomes, have a potentially underestimated role in inducing protective immunity both in primary infection as well as genetic vaccination against a human pathogen.

## Materials and Methods

### Ethics statement

This study was carried out in strict accordance with the recommendations in the Guide for the Care and Use of Laboratory Animals of the Brazilian National Council of Animal Experimentation (http://www.cobea.org.br/). The protocol was approved by the Committee on the Ethics of Animal Experiments of the Institutional Animal Care and Use Committee at the Federal University of Sao Paulo (Id # CEP 0426/09). The protocol using human samples was approved by the Institutional Review Board of the University of São Paulo School of Medicine (Protocol number 739/2005) and written informed consent was obtained from the patients. In the case of samples from heart donors, written informed consent was obtained from their families.

### Patients

Myocardial left ventricular free wall heart samples were obtained from end-stage heart failure chronic chagasic patients with cardiomyopathy (7 females, 7 males, 15–61 years old). Control adult heart tissue from the left ventricular-free wall was obtained from nonfailing donor hearts not used for cardiac transplantation due to size mismatch with available recipients (males, 17–46 years old). Hearts were explanted at the time of heart transplantation at the Heart Institute—InCor, University of São Paulo School of Medicine, São Paulo, SP, Brazil. For mRNA extraction, samples were quickly dissected, and myocardial tissue was frozen in liquid nitrogen and stored at -80°C.

### Mice and parasites

Five- to 8-week-old female C57BL/6 mice were purchased from CEDEME (Federal University of São Paulo). TKO mice were generated as described by Kincaid *et al*. [26] and were bred in our own animal facility. Bloodstream trypomastigotes of the Y or CL strain of *T*. *cruzi* were obtained from mice infected 7 or 15 days earlier. The concentration of parasites was estimated and adjusted to 10^5^ parasites/mL. Each mouse was inoculated with 10^4^ trypomastigotes diluted in 0.1 mL PBS administered subcutaneously (s.c.) at the base of the tail. Parasitemia was assessed on the days indicated in each figure, which involved counting the number of parasites per 5 μL blood.

### Genetic vaccination

The plasmid pIgSP Cl.9 and AdASP-2 were generated, grown and purified as described previously [28, 32]. Control mice were immunized with pcDNA3 and human replication-deficient adenovirus type 5 expressing βgal (Adβ-gal). The mice were inoculated intramuscularly (i.m.) with 50 μg plasmid DNA into each *tibialis anterioris* muscle. A total of 21 days later, these mice received 50 μL of a viral suspension containing 2 X 10^8^ plaque-forming units (pfu) of adenovirus via the same locations. Immunological assays were performed on the days indicated in each figure.

### Peptides

Synthetic peptides VNHRFTLV, ANYKFTLV, and ANYDFTLV were purchased from GenScript (Piscataway, NJ). The peptide purity was higher than 90%. Peptide identities were confirmed using a Q-Tof Micro equipped with an electrospray ionization source (Micromass, UK). The pentamer H-2K^b^-VNHRFTLV was purchased from ProImmune Inc. (Oxford, UK).

### BMDC generation

Progenitor cells were flushed from femurs and cultured *in vitro* in RPMI 1640 supplemented with 10 mM HEPES, 0.2% sodium bicarbonate, 59 mg/L penicillin, 133 mg/L streptomycin, 10% HyClone fetal bovine serum, 2 mM L-glutamine, 1 mM sodium pyruvate, 55 μM 2-mercaptoethanol and 20 ng/mL GM-CSF (R&D Systems) at a concentration of 2 X 10^5^ cells/mL. After 4 days in culture, half of the medium volume was replaced with fresh medium. At day 6, the resulting BMDCs were exposed to tissue-culture trypomastigotes of *T*. *cruzi* at a ratio of 3 parasites/cell or to AdASP-2 at a ratio of 50 pfu/cell for an additional 24 h. After 7 days in culture (and 24 h of *T*. *cruzi* or AdASP-2 exposure), the BMDCs were employed in *in vitro* antigen presentation assays.

### Immunological assays

The presence of IL-12p70 in the supernatant of the BMDCs was assessed after 7 days in culture (and 24 h of *T*. *cruzi* or AdASP-2 exposure) using an ELISA (BD).

To perform the *in vitro* antigen presentation assays, spleen cells from naïve animals or mice infected with *T*. *cruzi* 15 days earlier were harvested, and the CD8^+^ T cell population was isolated through negative selection with a CD8^+^ T Cell Isolation Kit using MACS beads (Miltenyi) according to the manufacturer’s specification, followed by staining with anti-CD8 PerCP (53–6.7, BD) and sorting by FACS (BD FACSAria II). The CD8^-^ fraction obtained after MACS isolation was stained with CD4 PE Cy7 (GK1.5, BD) and also sorted by FACS. The isolated lymphocytes were co-cultured with BMDCs previously exposed to *T*. *cruzi* or AdASP-2 or left unexposed at a ratio of 1 BMDC to 5 CD8 or CD4 cells for 24 h, and the number of IFN-γ-secreting cells was determined by ELISPOT, as described elsewhere [31].

For flow cytometry analyses, we used mouse splenocytes treated with ACK buffer (NH_4_Cl, 0.15 M; KHCO_3_, 10 mM; Na_2_-EDTA 0.1 mM; pH = 7.4) for lysing the erythrocytes. Single-cell suspensions were washed in PBS, stained for 10 min at RT with biotinylated MHC I multimer H-2K^b^-VNHRFTLV, and stained for 20 min at 4°C with streptavidin-APC and CD8 FITC (53–6.7). For the analyses of other cell-surface markers, single-cell suspensions from spleens of mice were stained with CD11c APCCy7 (HL3), CD86 APC (GL1), CD11b PerCP (M1/70), CD19 PE (ID3), CD3 PE (17A2), H-2K^b^ FITC (AF6-88.5), IAb biotinylated (25-9-17), streptavidin PECy7, CD4 PeCy7 (RM4-5), CD8 Pacific Blue (53–6.7), CD62L APC (MEL-14), and CD44 PE (IM7). Staining of TCR Vβ chains was performed using TCR Vβ Screening Panel Kit (BD Pharmingen). Isotype control antibodies were PE-labeled hamster IgG1k and rat IgG1k, and FITC-labeled IgG2ak. All antibodies and streptavidins were purchased from BD Pharmingen. At least 500,000 cells were acquired on a BD FACS Canto II flow cytometer and analyzed with FlowJo 8.7 (Tree Star, Ashland, OR).

For the intracellular staining (ICS) of cytokines (IFN-γ and TNF), splenocytes collected from C57BL/6 or TKO mice were treated with ACK buffer. ICS was performed after *in vitro* culture of splenocytes in the presence or absence of the peptides indicated in each figure. Cells were washed 3 times in plain RPMI and re-suspended in cell culture medium consisting of RPMI 1640 medium supplemented with 10 mM Hepes, 0.2% sodium bicarbonate, 59 mg/L of penicillin, 133 mg/L of streptomycin, 10% Hyclone fetal bovine serum, 2 mM L-glutamine, 1 mM sodium pyruvate, 55 μM 2-mercaptoethanol. The viability of the cells was evaluated using 0.2% trypan blue exclusion dye to discriminate between live and dead cells. Cell concentration was adjusted to 5 X 10^6^ cells/mL in cell culture medium containing anti-CD28 (2 μg/mL), BDGolgiPlug (1 μL/mL) and monensin (5 μg/mL). In half of the cultures, a final concentration of 10μM of the VNHRFTLV peptide was added. The cells were cultivated in V-bottom 96-well plates (Corning) in a final volume of 200 μL in duplicate, at 37°C in a humid environment containing 5% CO_2_. After 8h incubation, cells were stained for surface markers with CD4 FITC (GK1.5) and CD8 PerCP (53–6.7) on ice for 20 min. To detect IFN-γ and TNF by intracellular staining, cells were then washed twice in buffer containing PBS, 0.5% BSA, and 2 mM EDTA, fixed and permeabilized with BD perm/wash buffer. After being washed twice with BD perm/wash buffer, cells were stained for intracellular markers using APC-labeled anti-IFN-γ (XMG1.2) and PE-labeled anti-TNF (MP6-XT22) for 20 minutes on ice. Finally, cells were washed twice with BD perm/wash buffer and fixed in 1% PBS-paraformaldehyde. At least 800,000 cells were acquired on a BD FACS Canto II flow cytometer and then analyzed with FlowJo.

### Real-time PCR

Total RNA was isolated from mice and human myocardial tissues with Trizol (Invitrogen), followed by purification with Quick RNA Miniprep columns and DNAse I treatment (Zymo Research). cDNA was synthesized with High Capacity cDNA Reverse Transcription Kit (Applied Biosystems). To confirm the absence of genomic DNA control samples were used without reverse transcriptase. RT-PCR was performed using Power SYBR PCR Mastermix (Thermo Scientific) and StepOne Plus thermo cycler (Applied Biosystems). mRNA levels were normalized to HPRT (mouse) and beta-actin (human). Primer sequences are reported elsewhere for murine (procedure B in [48]) and human [49] samples. Relative quantification was calculated over *naïve* (mouse) or healthy (human) controls using Δ ΔCT method [50]. The protocol for quantification of *T*. *cruzi* DNA in heart and spleen samples was performed as described elsewhere [51].

### Bone marrow chimeras

Eight-week-old female C57BL/6 mice were irradiated at 900 Rads. Each irradiated animal received 10 × 10 ^6^ bone marrow cells *i*.*v*. isolated from C57BL/6 or TKO female mice. Before transfer, bone marrow cell suspensions were depleted of T cells with CD8a (Ly-2) MicroBeads and CD4 (L3T4) MicroBeads (Miltenyi Biotec). Mice were given medicated water containing sulfamethoxazole (200 mg/mL) and trimethroprim (40 mg/mL). Experiments were performed 8 weeks after bone marrow transfer.

### Statistical analysis

Groups were compared using One Way ANOVA followed by Tukey’s HSD test (http://faculty.vassar.edu/lowry/VassarStats.html). Parasitemia values were log transformed before comparison. The Log-rank (Mantel-Cox) test was used to compare mouse survival rates after challenge with *T*. *cruzi* (http://bioinf.wehi.edu.au/software/russell/logrank/). The differences were considered significant when the P value was <0.05.

## Supporting Information

S1 Fig *T*. *cruzi* MHC class I epitopes are processed through the cytosolic pathway. (a) TAP-1-deficient mice had their phenotype confirmed by the absence of CD8^+^ T cells in the spleen. (b) WT and TAP-1-deficient BMDC were incubated with LPS, *T*. *cruzi* or AdASP-2 and the expression of MHC and co-stimulatory molecules on CD11c^+^ cells was assessed by flow cytometry. These BMDC were co-cultured with (c) CD8^+^ or (d) CD4^+^ T cells isolated from the spleen of *T*. *cruzi*-infected mice and the ability to present antigen was assessed through ELISPOT to detect spot forming cells (SFC) secreting IFN-γ. (e) WT BMDC were incubated with *T*. *cruzi* or AdASP-2 in presence or absence of the proteasome inhibitor epoxomicin (EPO) 1 μM and the expression of MHC and co-stimulatory molecules on CD11c^+^ cells was assessed by flow cytometry. (f) These BMDC were co-cultured with CD8^+^ T cells isolated from the spleen of *T*. *cruzi*-infected mice and the ability to present antigen was assessed through ELISPOT to detect spot forming cells (SFC) secreting IFN-γ.
(TIF)

S2 Fig Reduced frequencies of CD8^+^ T cells in TKO animals. WT and TKO mice were infected *s*.*c*. with 10^4^ *T*. *cruzi* parasites or left uninfected. Frequencies of (a) CD8^+^ and (b) CD4^+^ T cells in the spleen of these animals are shown. Results are expressed as individual values and the mean ± SEM for each group. Asterisks indicate that the values observed for TKO mice were significantly lower than those for WT mice (*P<0.05 ***P<0.001).
(TIF)

S3 Fig Impaired immunity of CD8^+^ T cells in TKO animals infected with *T*. *cruzi*. WT and TKO mice were infected *s*.*c*. with 10^4^ *T*. *cruzi* parasites or left uninfected. Twenty days later, the response of CD8^+^ T cells was assessed in the spleen. (a) Frequencies of CD8^+^ CD44^high^ CD62L^low^ cells. (b) Frequencies of specific CD8^+^ T cells stained with H-2K^b^-VNHRFTLV pentamers. (c) Frequencies of CD8^+^ splenic cells positively stained with anti-TNF and/or anti-IFN-γ after *ex vivo* restimulation with the indicated peptides corresponding to known or hypothetical *T*. *cruzi* MHC class I-restricted epitopes. (d) Numbers of spot forming cells (SFC) secreting IFN-γ and (e) representative samples from ELISPOT of spleen cells upon restimulation with the indicated peptides. Results are shown as individual values and as the mean ± SEM for each group. Asterisks indicate that the values observed for TKO mice were significantly lower than those for WT mice (*P<0.05 **P<0.01 ***P<0.001 ****P<0.0001).
(TIF)

S4 Fig Unaltered immunity mediated by CD4^+^ T cells in TKO animals infected with *T*. *cruzi*. WT and TKO mice were infected *s*.*c*. with 10^4^ *T*. *cruzi* parasites or left uninfected. Twenty days later, their spleens were collected and the frequencies of (a) CD4^+^ CD44^high^ CD62L^low^ cells and (b) CD4^+^ T cells producing IFN-γ and/or TNF were estimated by intracellular staining. The results are expressed as individual values and as the mean ± SEM for each group.
(TIF)

S5 Fig Impaired immunity of CD8^+^ T cells in TKO animals genetically vaccinated against *T*. *cruzi*. WT and TKO mice were primed with empty plasmid DNA (pcDNA3) or a plasmid vector expressing ASP-2 (pIgCl9) and boosted after 21 days with adenovirus 5 expressing beta-galactosidase (Adβ-gal) or ASP-2 (AdASP-2), respectively. Fifteen days later, the response of CD8^+^ T cells was assessed in the spleen. (a) Frequencies of specific CD8^+^ T cells stained with H-2K^b^-VNHRFTLV pentamers. (b) Frequencies of CD8^+^ splenic cells positively stained with anti-TNF and/or anti-IFN-γ after *ex vivo* restimulation VNHRFTLV peptide. (c) Numbers of spot forming cells (SFC) secreting IFN-γ detected by ELISPOT of spleen cells upon restimulation with the peptide VNHRFTLV. Results are shown as individual values and as the mean ± SEM for each group. Asterisks indicate that the values observed for TKO mice were significantly lower than those for WT mice (****P<0.0001).
(TIF)

S6 Fig Unaltered response of CD4^+^ T cells in TKO animals genetically immunized with A*sp-2*. WT and TKO mice were immunized with plasmid DNA encoding *Asp-2* and boosted after 21 days with the viral vector AdASP-2. Following immunization, mice were given 2 mg BrdU i.p. every other day. Fifteen days after boost, their spleens were collected and the frequencies of CD8^+^ CD44^high^ BrdU^+^ and CD4^+^ CD44^high^ BrdU^+^ cells were determined by flow cytometry. These results are expressed as individual values and as the mean ± SEM for each group (n = 3). Asterisks indicate that the values observed for TKO mice were significantly lower than those for WT mice (*P<0.05). Alternatively, splenocytes from WT and TKO immunized mice were re-stimulated *ex vivo* with AdASP-2-infected BMDC followed by IFN-γ staining in CD4^+^ and CD8^+^ cells.
(TIF)

S7 Fig Susceptibility of TKO animals to challenge with CL strain of *T*. *cruzi*. WT and TKO mice were challenged intraperitoneally with 100 bloodstream parasites. (a) Parasitemia at different time points after infection. (b) Mortality rates after challenge. The parasitemia values were compared by one-way ANOVA, which showed that WT mice displayed levels of parasitemia that were significantly lower than those of TKO animals (p<0.01 in all cases). Comparison of the survival curves using the log-rank (Mantel-Cox) test indicated that WT mice survived significantly longer (p<0.01) than TKO mice did.
(TIF)
