# Supplementary figures and images for: The Combined Deficiency of Immunoproteasome Subunits Affects Both the Magnitude and Quality of Pathogen- and Genetic Vaccination-Induced CD8+ T Cell Responses to the Human Protozoan Parasite Trypanosoma cruzi
Source: PLoS Pathog. 2016 Apr 29;12(4):e1005593. doi: 10.1371/journal.ppat.1005593 (PMC4851296; doi:10.1371/journal.ppat.1005593)

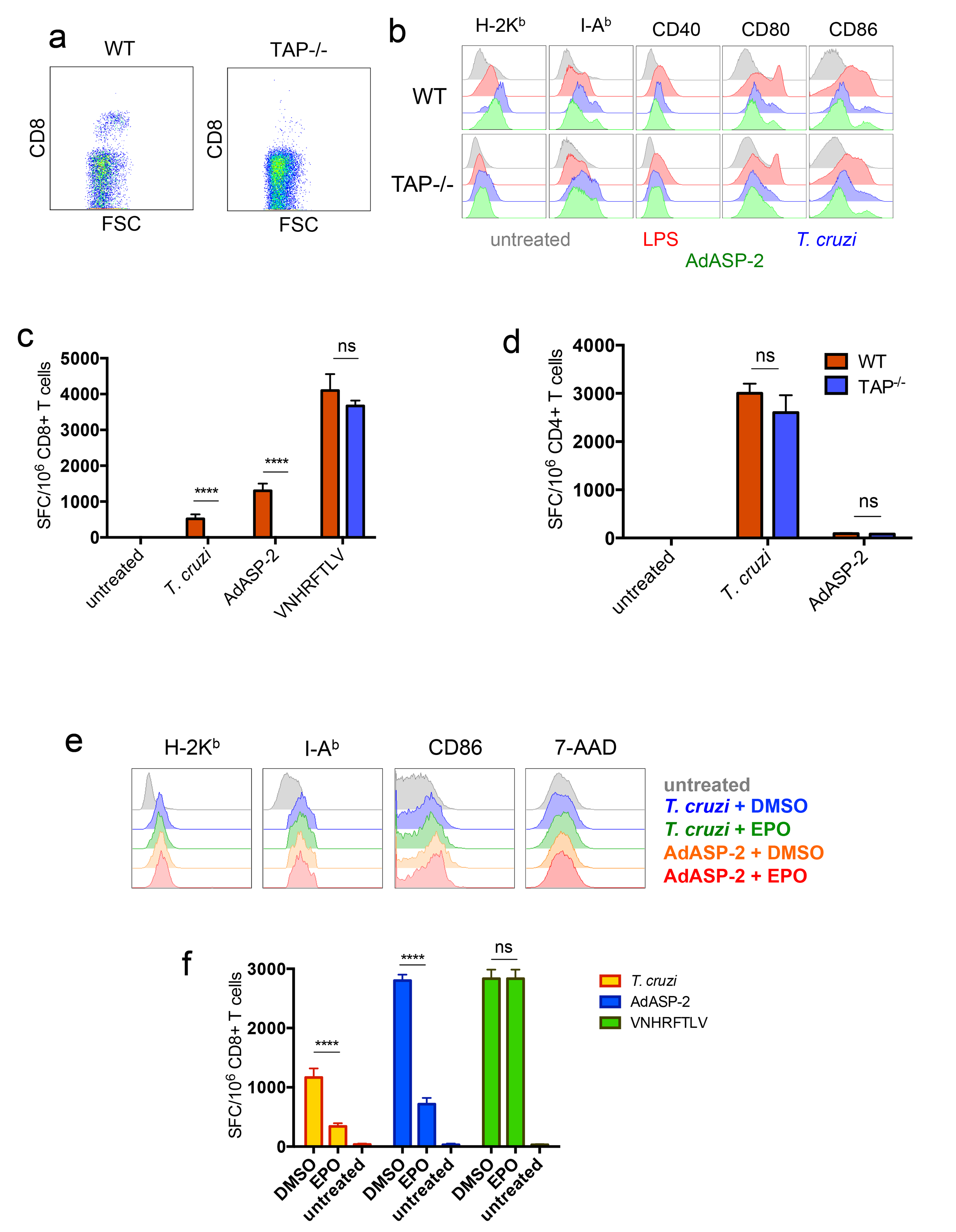

Supplement: S1 Fig — (a) TAP-1-deficient mice had their phenotype confirmed by the absence of CD8+ T cells in the spleen. (b) WT and TAP-1-deficient BMDC were incubated with LPS, T. cruzi or AdASP-2 and the expression of MHC and co-stimulatory molecules on CD11c+ cells was assessed by flow cytometry. These BMDC were co-cultured with (c) CD8+ or (d) CD4+ T cells isolated from the spleen of T. cruzi-infected mice and the ability to present antigen was assessed through ELISPOT to detect spot forming cells (SFC) secreting IFN-γ. (e) WT BMDC were incubated with T. cruzi or AdASP-2 in presence or absence of the proteasome inhibitor epoxomicin (EPO) 1 μM and the expression of MHC and co-stimulatory molecules on CD11c+ cells was assessed by flow cytometry. (f) These BMDC were co-cultured with CD8+ T cells isolated from the spleen of T. cruzi-infected mice and the ability to present antigen was assessed through ELISPOT to detect spot forming cells (SFC) secreting IFN-γ. (TIF) [file ppat.1005593.s001.tif]

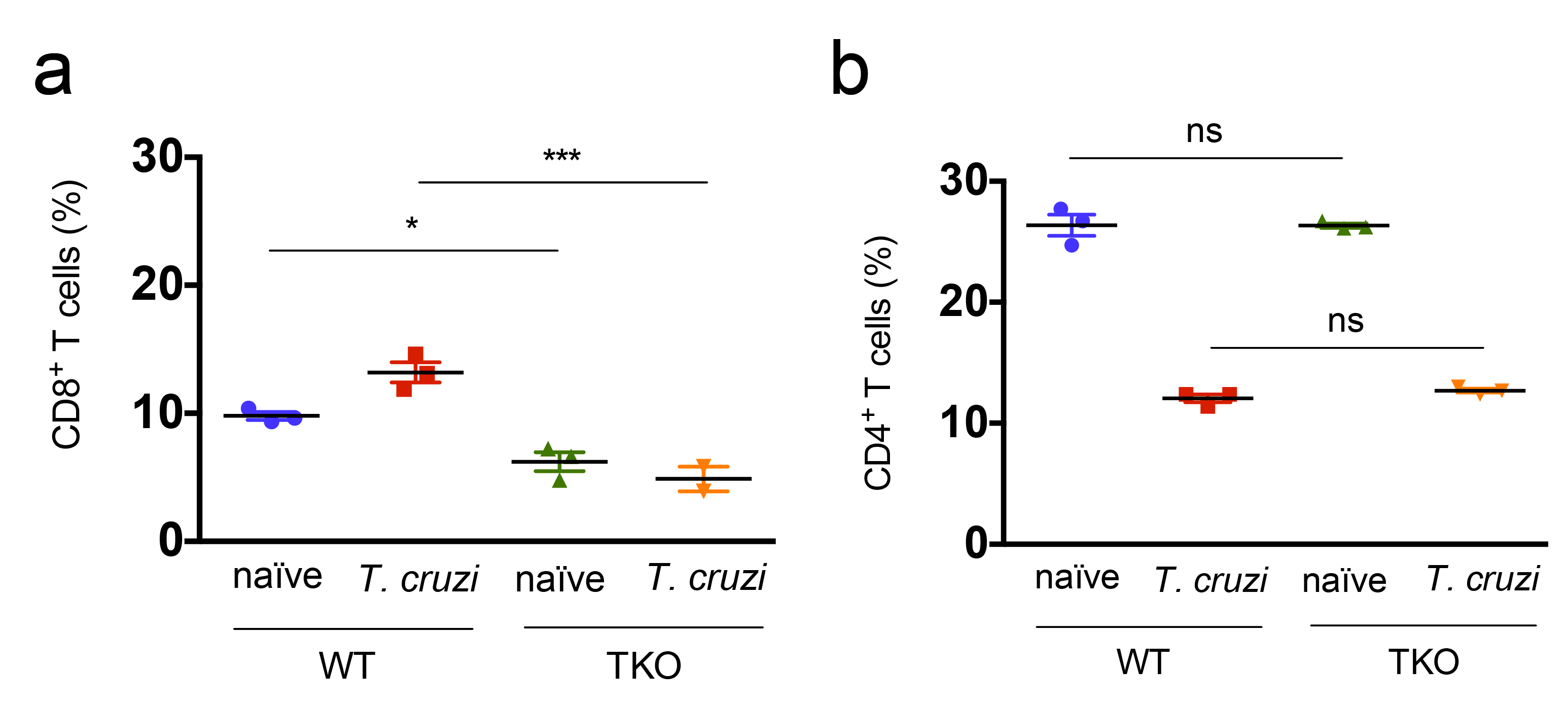

Supplement: S2 Fig — WT and TKO mice were infected s.c. with 104 T. cruzi parasites or left uninfected. Frequencies of (a) CD8+ and (b) CD4+ T cells in the spleen of these animals are shown. Results are expressed as individual values and the mean ± SEM for each group. Asterisks indicate that the values observed for TKO mice were significantly lower than those for WT mice (*P<0.05 ***P<0.001). (TIF) [file ppat.1005593.s002.tif]

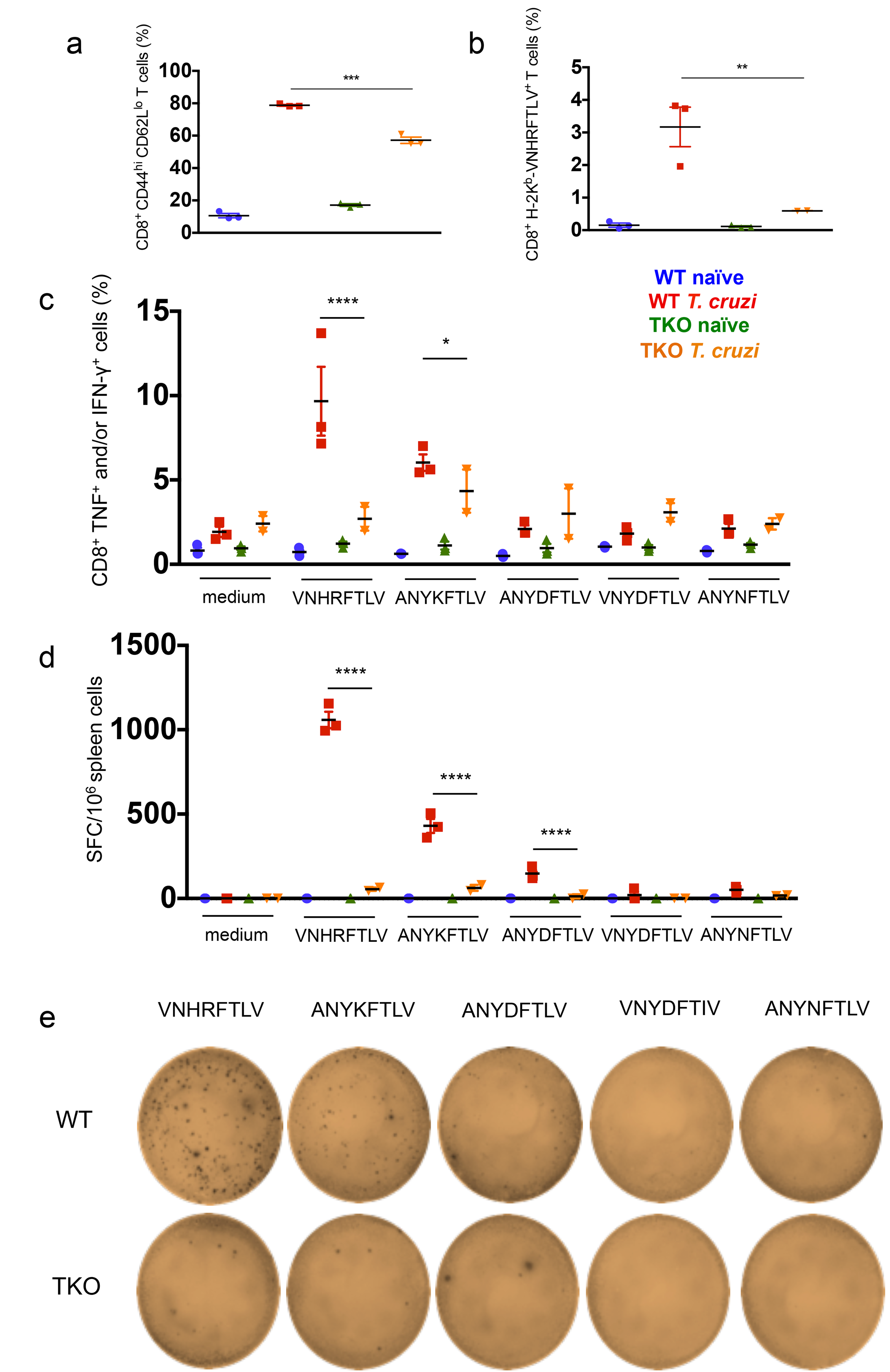

Supplement: S3 Fig — WT and TKO mice were infected s.c. with 104 T. cruzi parasites or left uninfected. Twenty days later, the response of CD8+ T cells was assessed in the spleen. (a) Frequencies of CD8+ CD44high CD62Llow cells. (b) Frequencies of specific CD8+ T cells stained with H-2Kb-VNHRFTLV pentamers. (c) Frequencies of CD8+ splenic cells positively stained with anti-TNF and/or anti-IFN-γ after ex vivo restimulation with the indicated peptides corresponding to known or hypothetical T. cruzi MHC class I-restricted epitopes. (d) Numbers of spot forming cells (SFC) secreting IFN-γ and (e) representative samples from ELISPOT of spleen cells upon restimulation with the indicated peptides. Results are shown as individual values and as the mean ± SEM for each group. Asterisks indicate that the values observed for TKO mice were significantly lower than those for WT mice (*P<0.05 **P<0.01 ***P<0.001 ****P<0.0001). (TIF) [file ppat.1005593.s003.tif]

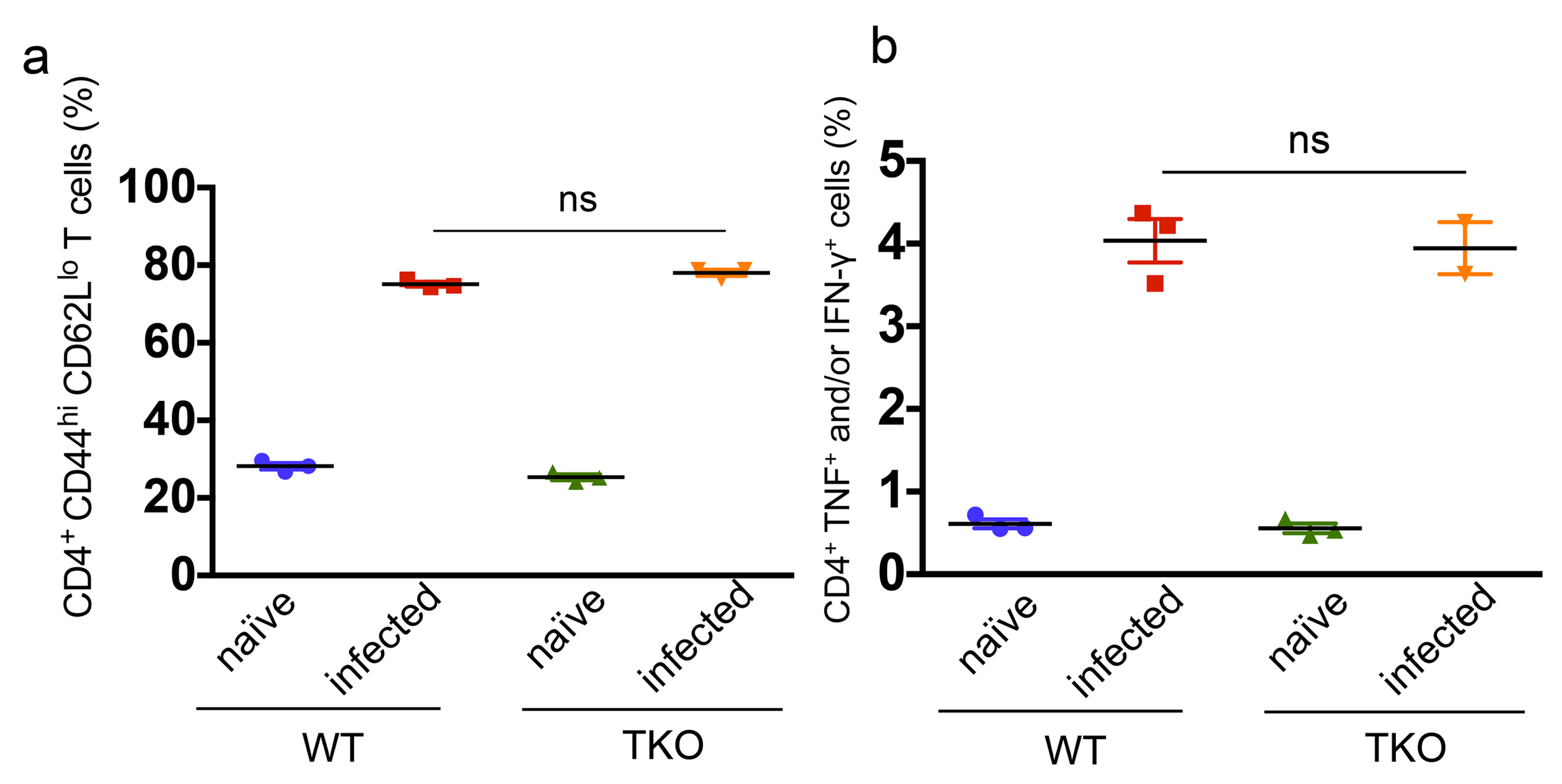

Supplement: S4 Fig — WT and TKO mice were infected s.c. with 104 T. cruzi parasites or left uninfected. Twenty days later, their spleens were collected and the frequencies of (a) CD4+ CD44high CD62Llow cells and (b) CD4+ T cells producing IFN-γ and/or TNF were estimated by intracellular staining. The results are expressed as individual values and as the mean ± SEM for each group. (TIF) [file ppat.1005593.s004.tif]

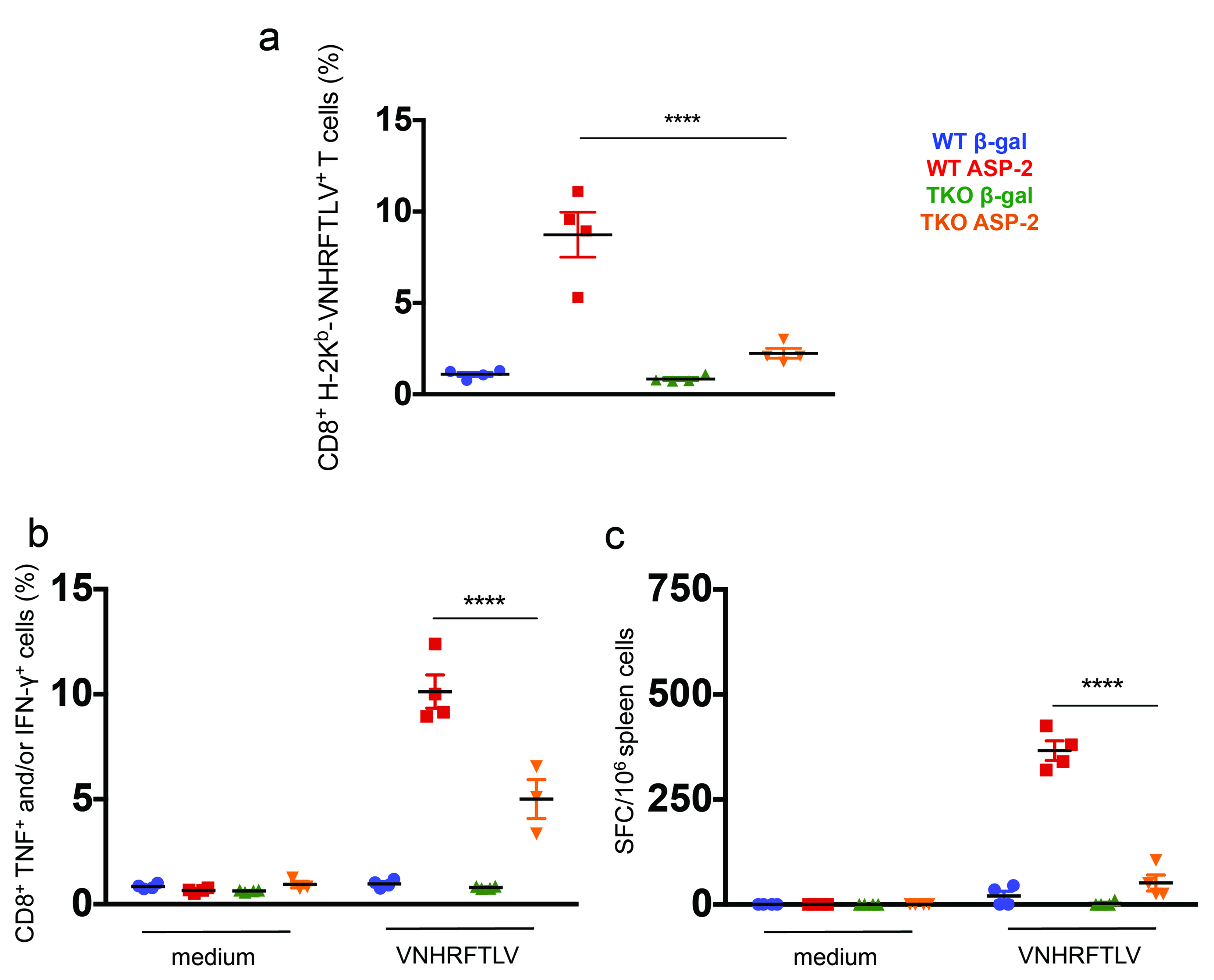

Supplement: S5 Fig — WT and TKO mice were primed with empty plasmid DNA (pcDNA3) or a plasmid vector expressing ASP-2 (pIgCl9) and boosted after 21 days with adenovirus 5 expressing beta-galactosidase (Adβ-gal) or ASP-2 (AdASP-2), respectively. Fifteen days later, the response of CD8+ T cells was assessed in the spleen. (a) Frequencies of specific CD8+ T cells stained with H-2Kb-VNHRFTLV pentamers. (b) Frequencies of CD8+ splenic cells positively stained with anti-TNF and/or anti-IFN-γ after ex vivo restimulation VNHRFTLV peptide. (c) Numbers of spot forming cells (SFC) secreting IFN-γ detected by ELISPOT of spleen cells upon restimulation with the peptide VNHRFTLV. Results are shown as individual values and as the mean ± SEM for each group. Asterisks indicate that the values observed for TKO mice were significantly lower than those for WT mice (****P<0.0001). (TIF) [file ppat.1005593.s005.tif]

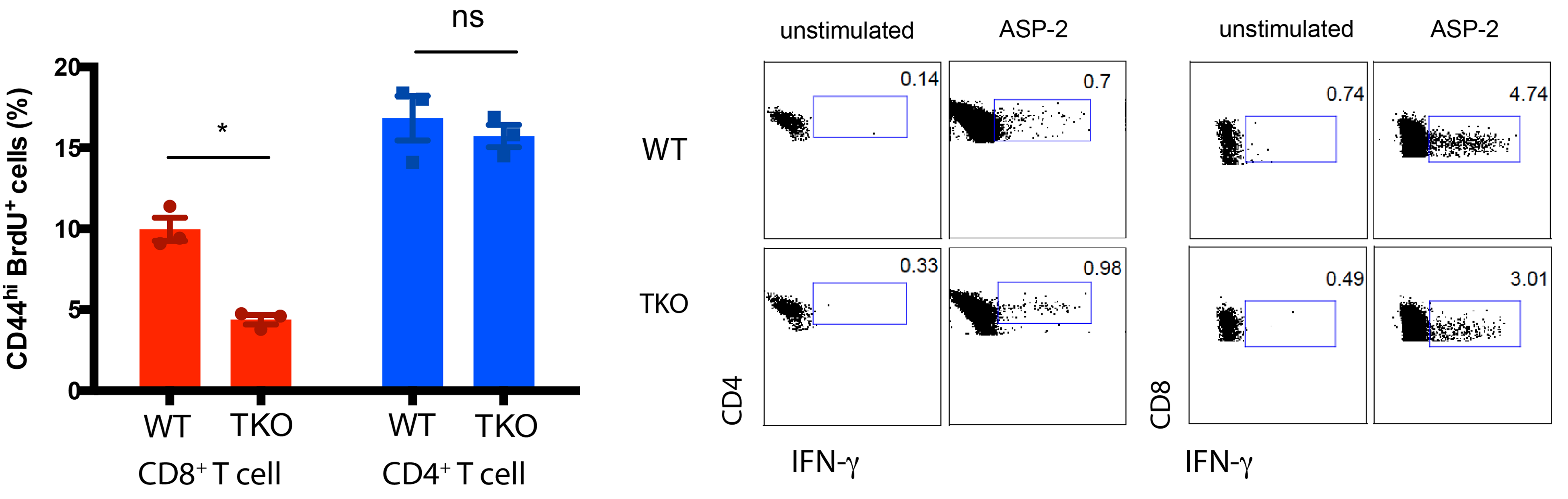

Supplement: S6 Fig — WT and TKO mice were immunized with plasmid DNA encoding Asp-2 and boosted after 21 days with the viral vector AdASP-2. Following immunization, mice were given 2 mg BrdU i.p. every other day. Fifteen days after boost, their spleens were collected and the frequencies of CD8+ CD44high BrdU+ and CD4+ CD44high BrdU+ cells were determined by flow cytometry. These results are expressed as individual values and as the mean ± SEM for each group (n = 3). Asterisks indicate that the values observed for TKO mice were significantly lower than those for WT mice (*P<0.05). Alternatively, splenocytes from WT and TKO immunized mice were re-stimulated ex vivo with AdASP-2-infected BMDC followed by IFN-γ staining in CD4+ and CD8+ cells. (TIF) [file ppat.1005593.s006.tif]

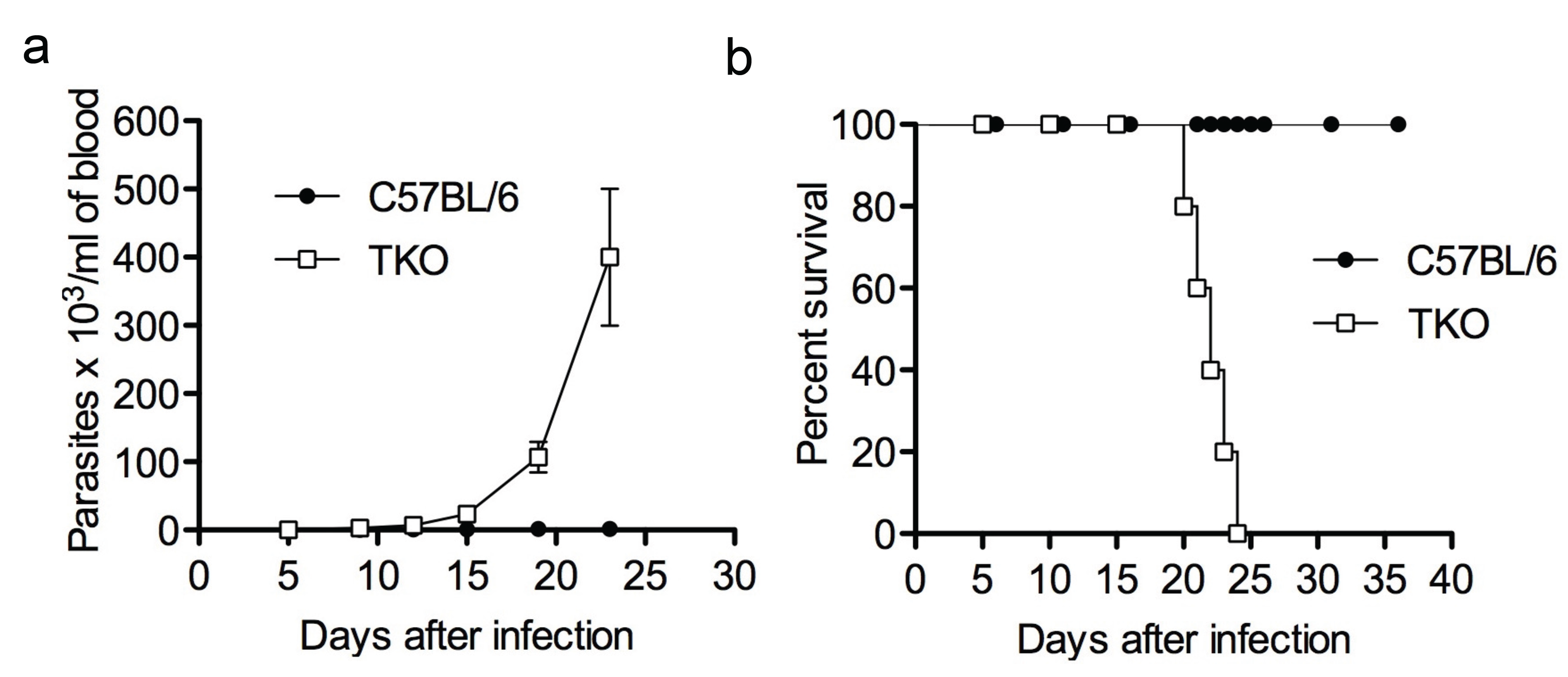

Supplement: S7 Fig — WT and TKO mice were challenged intraperitoneally with 100 bloodstream parasites. (a) Parasitemia at different time points after infection. (b) Mortality rates after challenge. The parasitemia values were compared by one-way ANOVA, which showed that WT mice displayed levels of parasitemia that were significantly lower than those of TKO animals (p<0.01 in all cases). Comparison of the survival curves using the log-rank (Mantel-Cox) test indicated that WT mice survived significantly longer (p<0.01) than TKO mice did. (TIF) [file ppat.1005593.s007.tif]
